# Insights Gained From a Re-analysis of Five Improvement Cases in Healthcare Integrating System Dynamics Into Action Research

**DOI:** 10.34172/ijhpm.2022.5693

**Published:** 2022-02-26

**Authors:** Paul Holmström, Thomas Björk- Eriksson, Pål Davidsen, Fredrik Bååthe, Caroline Olsson

**Affiliations:** ^1^Department of Clinical Radiation Sciences, Institute of Clinical Sciences, Sahlgrenska Academy, Gothenburg University, Gothenburg, Sweden.; ^2^Regional Cancer Centre West, Gothenburg, Sweden.; ^3^Department of Oncology, Institute of Clinical Sciences, Sahlgrenska Academy, Gothenburg University, Gothenburg, Sweden.; ^4^Department of Geography, University of Bergen, Bergen, Norway.; ^5^LEFO – Institute for Studies of the Medical Profession, Oslo, Norway.; ^6^Institute of Stress Medicine, Gothenburg, Sweden.; ^7^Sahlgrenska University Hospital, Gothenburg, Sweden.; ^8^Institute of Health and Care Sciences, Sahlgrenska Academy, Gothenburg University, Gothenburg, Sweden.

**Keywords:** Simulation, Implementation, Mixed Methods, System Dynamics, Action Research, Healthcare

## Abstract

**Background:** Healthcare is complex with multi-professional staff and a variety of patient care pathways. Time pressure and minimal margins for errors, as well as tension between hierarchical power and the power of the professions, make it challenging to implement new policies or procedures. This paper explores five improvement cases in healthcare integrating system dynamics (SD) into action research (AR), aiming to identify methodological aspects of how this integration supported multi-professional groups to discover workable solutions to work-related challenges.

**Methods:** This re-analysis was conducted by a multi-disciplinary research group using an iterative abductive approach applying qualitative analysis to structure and understand the empirical material. Frameworks for consultancy assignments/client projects were used to identify case project stages (workflow steps) and socio-analytical questions were used to bridge between the AR and SD perspectives.

**Results:** All studied cases began with an extensive AR-inspired inventory of problems/objectives and ended with an SD-facilitated experimental phase where mutually agreed solutions were tested *in silico*. Time was primarily divided between facilitated group discussions during meetings and modelling work between meetings. Work principles ensured that the voice of each participant was heard, inspired engagement, interaction, and exploratory mutual learning activities. There was an overall pattern of two major divergent and convergent phases, as each group moved towards a mutually developed point of reference for their problem/objective and solution, a case-specific multi-professional knowledge repository.

**Conclusion:** By integrating SD into AR, more favourable outcomes for the client organization may be achieved than when applying either approach in isolation. We found that SD provided a platform that facilitated experiential learning in the AR process. The identified results were calibrated to local needs and circumstances, and compared to traditional top-down implementation for change processes, improved the likelihood of sustained actualisation.

## Background

**Key Messages**

** Implications for policy makers**

- The described work principles lead to higher probability of actualization of developed solutions. Simulations also provided reality-checks, increasing the probability that solutions will work in practise.
- Integrating system dynamics (SD) into action research (AR) can be time efficient as potential solutions can be tested *in silico* prior to testing in practise.
- The work principles ensured that the participants went beyond stakeholderism as they saw how their perspectives fitted in with those of others. Deep engagement of participants, co-creation, and experiential learning through simulations lead to convergence of mindsets.

** Implications for the public**

The healthcare sector has needs for change to adapt to new procedures, medications, and technologies, but with little time and resources to plan and implement change. Workloads are high and there is often resistance to abandon routines that work. Any change of policies or procedures requires that different professions work together to define new routines. Many change initiatives are perceived as top-down and as lacking understanding of local conditions. Also “quick fixes” may lead to unintended consequences as they do not consider the wider system. The methods presented here are time-efficient and allow for local adaptation of general principles, while creating engagement and commitment across professional and departmental boundaries.

Healthcare is complex and is characterized by a variety of patients and care pathways as well as multi-professional staff serving these pathways. Time pressure and minimal margins for errors make it challenging to implement new policies or procedures, no matter how desirable they are.^1-3^ May et al^4^ suggest using questionnaires to assess organisational preparedness for top-down implementation, whereas Øvretveit et al^5^ and Bååthe and Norbäck^6^ describe the futility in trying to define context-independent principles of implementation, by referring to the observation that any solution in healthcare needs to be calibrated to local needs and circumstances. The tension between the hierarchical organisation and the professions as described by Mintzberg^7^ may lead to mistrust and powerplay,^8^ further complicating change efforts.

System dynamics (SD) offers methods, techniques, and tools that are intended to bring about a shared understanding of reality and its systemic implications. An SD-based modelling process usually encompasses: (1) problem articulation, (2) formulation of a dynamic hypothesis, (3) formulation of a simulation model, (4) model testing, and (5) policy design and evaluation.^9^ SD requires expertise in the method, as well as in the use of associated software tools. SD simulations allow for the rapid development, testing, and reality-checking of scenarios for coherence and consistency *in silico* before implementing solutions in real life. Group model building (GMB) is often used in SD. Rouwette^10^ describes how GMB leads to the convergence of mental models (ie, minds) through mutual information exchange between participants in GMB sessions. This convergence is also described as stakeholder alignment (to consolidate potentially competitive perspectives).^11^ Voinov et al^12^ further describe tools and methods that can be used at different stages of GMB and Holmström and Elf have scoped group interventions that may be suitable to use with GMB.^13^ GMB is usually carried out by using prepared sequenced steps in workshops, so called scripted GMB, where participants often are expected to learn the basics of model building and SD terminology.^14-17^ This can pose a challenge for participants, but on the other hand, scripted GMB enables SD modellers less experienced in facilitation to lead workshops.

Action research (AR) is a field of research that is based on actively engaging participants that are willing to share their own perspective on a problem, to collaborate so as to find a mutually acceptable solution to that problem and then to, pragmatically, investigate how this solution actually works in practice.^18^ The process of AR is usually carried out in cycles of Plan, Do, Study, Act, where participants take informed decisions about how to proceed based on collected data and experience from a previous cycle.^19^ In healthcare, AR projects are typically specific to the work context of the participants, focus on their perceived problems, and is oriented towards developing their workplace here and now. People working together meet in order to identify and address a problem and to generate and assess alternative improvement; they iteratively reflect on their current practices, identify possible actions, and then try them out in order to learn from reality. The AR process also leads to shared mental models, but in contrast to SD, these remain as mindsets and are not necessarily explicitly stated in formal models. Consequently, solutions are derived and assessed in reality, which often makes an AR process time consuming. AR is based on deep participant engagement and a clear sense of ownership of the problems as well as their resolutions. “Implementation” is a concept rarely used in the AR literature. Rather AR relies on shared energy in the group that leads to actualization. If the results obtained are not as expected, then the group asks “what have we missed” and works through yet another iteration to find a more functional solution to try out, – a process not unlike the reality checks applied in SD. The process continues until a satisfactory result is reached.

Mingers and Gill^20^ suggest that no single methodology can offer a complete view of the complexities facing organisations and their members and suggest that the application of two or more methodologies are more likely to produce a realistic representation of the challenges facing them, which in turn, leads to better decisions. Hesse-Biber^21^ suggests that mixed methods forges new pathways and provides innovation. Zolfagharian et al^22^ found that there is little knowledge about why, when, and how SD is combined with any particular method. Moreover, Howick and Ackermann^23^ similarly conclude that there appears to be little discussion about generic lessons from mixing methods in practice. Since simulation-based analysis allows for timely, *in silico* testing, and rejection or acceptance of proposed solutions, time may be saved for such an approach in comparison to a process whereby the assessment of solutions takes place in reality such as in pure AR. Keeping in mind, however, that actualization rates of SD interventions have been reported to be around 5%, in spite of the fact that the interventions proposed appear to yield beneficial results *in silico*,^24,25^ identifying strategies which can increase chances of actualizing identified solutions are needed. Scholl^26^ describes how both SD and AR have iterative processes and suggests that what participants learn through SD simulations can be applied in the reality and what they learn in the real world can improve the virtual reality of the simulations. Scholl proposes that SD can facilitate every phase of AR. Little has, however, been reported about experiences from integrating any simulation method into an AR process.^27-29^

 Here we analyse five improvement cases in healthcare where SD was integrated into AR processes to support multi-professional groups in finding solutions to work-related challenges. The aim of our study was to explore how this particular integration adapted to each case over time and to identify related work patterns and principles which can provide methodological guidance for practitioners and improve chances of actualisation in the client organization.

## Materials and Methods

###  Materials – the Studied Cases 

**Figure 1:**
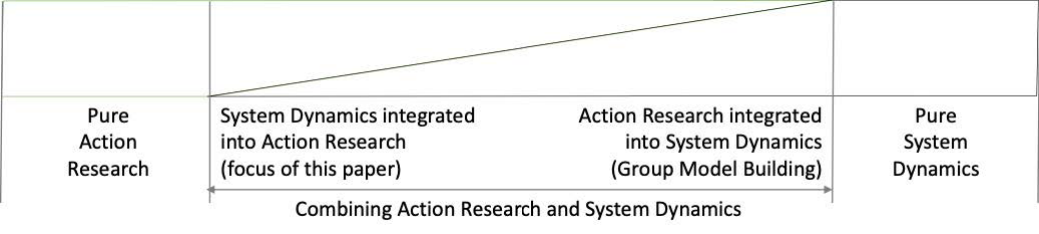
Schematic of Possible Degrees of Mix Between Action Research and System Dynamics From a Mixed-Methods Perspective. In contrast to the scientific literature about AR, GMB (where AR is integrated into SD), and SD, integrating SD into AR has to date been little studied. Abbreviations: AR, action research; SD, system dynamics; GMB, group model building.

Five improvement cases in healthcare, carried out over seven years (2004-2011) were studied. A summary of case characteristics is given below, and a detailed overview of each case is given in Supplementary file 1. All cases were selected from the case experience of the first author (the main facilitator and modeller with extensive case experience, from pure AR, from pure SD, as well as from combined approaches) and were included in the re-analysis based on two selection criteria: (1) having addressed change and improvement processes in healthcare including multi-professional groups, (2) having been executed using SD integrated into AR as a choice of method to suggest and study potentially actionable solutions to problems/objectives posed (Figure 1).

 All cases originated from Swedish healthcare, national population of 10.4 million inhabitants, where 21 regional councils and 290 municipalities manage and provide healthcare. Municipalities are mainly responsible for elderly care and regional councils are responsible for primary care as well as special care. Case 2 was carried out in a small municipality, the other cases in medium to large regional hospitals, catchment areas between 260 000 and 2 000 000 inhabitants. Each of the five cases was carried out within a time frame of 4-6 months with 4-5 group meetings and planning and modelling work in-between. Implementation of solutions in reality was not within the initial scope of any of the cases. The studied groups were composed of members of different professions in healthcare.

 Case 1 concerned a stroke ward, where there was a perceived need of additional patient beds and a wish to determine qualitative factors for improving patient survival and health status when their medical treatment was completed. Case 2 was an obstetrics department, where staff and patients were dissatisfied with current scheduling practices. New work practices had been identified that potentially could solve the issues. However, there was strong uncertainty about how to apply those principles without worsening the situation. Case 3 was a dementia care home that was preparing a reorganisation for patient-centred care as well as planning for the adaptation of the premises to modern practices of dementia care. Case 4 involved a paediatrics department where premises were too cramped in periods with high levels of infectious diseases and were expected to be insufficient with respect to number of patients due to the closure of a satellite unit and increased child population. Case 5 concerned an accident and emergencies department with premises crowded by patient flow peaks several times per week to levels where staff perceived loss of full control and risks for patient safety. The department wanted to review work practices prior to planning for new or rebuilt premises.

###  Method – Case Re-analysis

 The re-analysis of the cases was conducted by researchers from several disciplines, all providing different and complementing perspectives. The first author was accompanied by two professors (SD and clinical sciences, respectively), one associate professor (clinical sciences, experienced in information sciences in healthcare) and one PhD (health and care sciences, experienced in AR in healthcare). In the following, we define a work pattern as how steps in a process may be sequenced or related to each other, whereas a work principle is valid throughout an entire process.

####  Data Processing 

All field notes, e-mails and models were revisited and summarized through four major iterations of analysis, using displays according to the methodologies suggested by Miles, Huberman and Saldana^30^ and by Eisenhardt.^31^ The analytical process is described in Supplementary file 2. The approach was abductive, where each iteration began with a provisional adoption of an explanatory hypothesis^32^ Each analysis was first carried out individually by each co-author, then in group discussions, comparing and contrasting individual observations. This was repeated until the authors arrived at condensed and coherent descriptions of the material.

####  Project Stages

To understand the development of each case, we used a modification of Kubr’s five steps and James and colleagues’ five approaches to consultancy assignments and client projects. Kubr^33^ describes the scope of consultancy assignments as consisting of five stages: entry, diagnosis, action planning, implementation, and termination. James et al^34^ takes a skills-based approach and describes five approaches to the client project: statistical analysis, modelling of key variables, problem identification, implementation of solutions or to be a sounding board, and to select a consultant with matching skills. We analysed the cases by five stages: diagnosis of problems, analysis of facts, modelling of key variables, action planning, and implementation. We excluded Kubr’s entry and termination as not being part of the processes investigated here and James and colleagues’ sounding board as being a different type of consultancy work. We also carried out bottom-up analysis of each case to uncover and describe workflows by case and define generalized workflow steps. In addition, the implementation stage was structured using Brailsford’s^24^ three levels of implementation of simulations. These are categorized in *suggested* (theoretically proposed by the modellers), *conceptualised* (discussed with a client organisation), and *implemented* (actually used in practice). This helped to quantify the degree of implementation and identify potential barriers to immediate implementation.

####  System Dynamics, Action Research, and Mixed-Methods Perspectives 

To understand the overall mixed-method process, the cases were analysed through an AR perspective, using Rowbottom’s^35^ four questions regarding what is known, assumed, extant, and requisite about the work situation at each stage of the process, supplemented by corresponding interpretations in the SD context to further understand how the cases moved forward (Supplementary file 3). The framework is an enrichment of Rowbottom’s questions and provides an AR-based structure to study the interaction between AR and SD when comparing the five cases and drawing conclusions regarding general work patterns and principles. The share of time between facilitation and modeling work was estimated in each case.

## Results

###  Work Patterns

####  Adaptive Workflows

**Figure 2:**
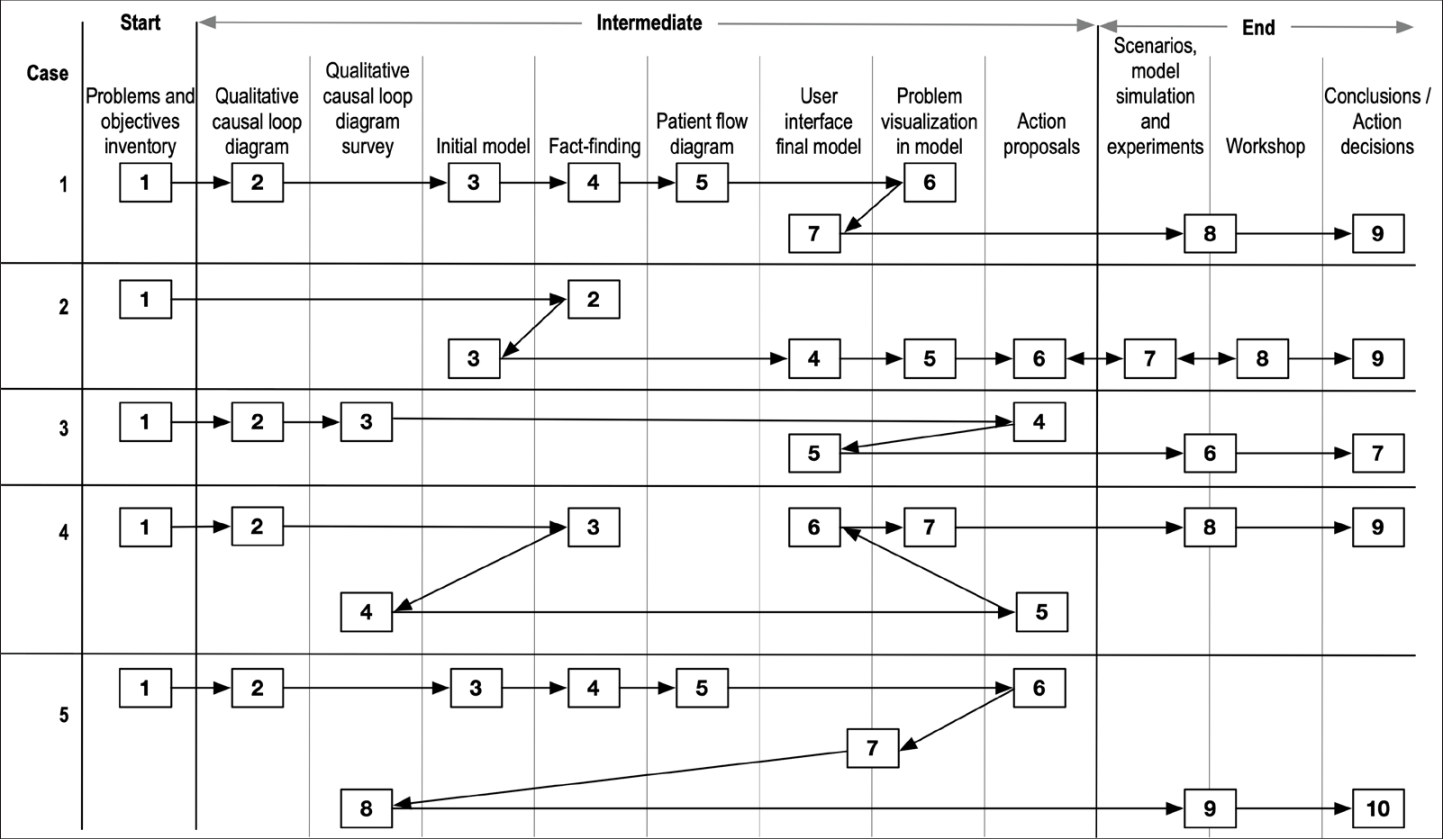
Chronological Workflows by Case. All cases began with an extensive facilitated problems and objectives inventory, starting with individual reflection and notes, then displayed to the group. The processes were purposively exhaustive so that all participants were satisfied that everything had been listed. Intermediary steps were sequenced differently between cases, based on pragmatic judgements regarding what would best move the process forwards. At all steps, facilitation was carried out to ensure that participants owned the issues, not the modeller. All cases ended in a workshop with an experimental part where a multitude of solutions were tested *in silico.*

The workflows by case are presented chronologically in Figure 2. The headings in the figure reflects work carried out either in a group, by the modeller, or by both. All cases had the same beginning and end, but had different sequences of intermediate steps.

####  Development of Solutions by Case

**Figure 3:**
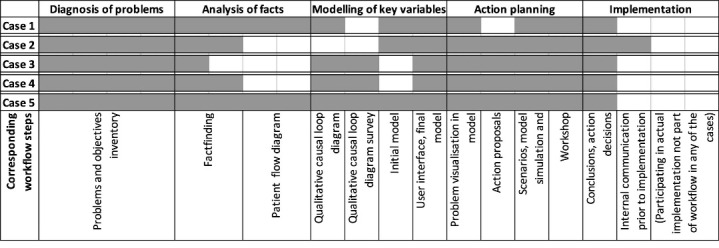
The Modeller’s Participation by Project Stage According to the Modification of Kubr and James et al and the Identified Workflow Steps. Note that participation in actual implementation was not included in the scope of any of the cases. However, in Case 2, the modellers participated in the introduction of the solution to the staff prior to the implementation and were invited to the final evaluation.

Figure 3 shows the modeller’s work by case using the modification of Kubr^33^ and James et al.^34^ In all cases, the groups worked through a diagnosis of problems, an analysis of facts and modelling of key variables and their interaction (ie, the systems structure). Differences between cases were primarily found in the parts relating to analysis of facts and modelling of key variables.

####  Divergent and Convergent Phases Towards a Multi-professional Knowledge Repository

**Figure 4:**
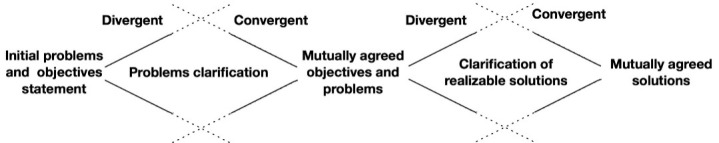
The Two Divergent and Convergent Phases. The starting point of all processes was an initial problems statement, which in a first phase was extended in a divergent and creative process ensuring that all issues were under discussion, and which then was converged by prioritization into a set of mutually agreed problems. The second phase began with divergent/creative work, proposing a variety of solutions, which were tested in the simulation, and which then was converged into a set of mutually agreed solutions to potentially be implemented in reality.

All cases were characterized by the same kind of oscillating pattern in the group processes. The processes were divergent and creative when actively stimulating the raising and listing of a variety of problems or suggested solutions. Convergent, when trying to reach consensus, prioritizing and agreeing on what to focus on. All groups went through two major phases of divergence and convergence (Figure 4).

During the first divergent phase, problems and objectives were listed until “everything was on the table” and mutually agreed upon. Instead of participants acting as stakeholders, they were encouraged to work as if they were laying out a jigsaw puzzle, exploring how each person’s knowledge and skills complemented that of the others so as to effectively address the problem and context at hand. Over time, this process resulted in their *multiprofessional knowledge repository,* a shared point of reference, which led to deeper individual and collective understanding of what went on in the system associated with the problem they studied.

 During the second divergent phase, suggestions of possible solutions and policies were encouraged. The simulation model and user interface were adapted to allow for the testing of a multitude of suggestions and clarified what could work in reality. During this phase, the group again converged, this time on a mutually agreed distilled number of solutions or policies which formed their proposal for how to continue. Thus, their multiprofessional knowledge repository became consolidated.

###  Work Principles

####  Studying the Interaction of Integrating System Dynamics Into Action Research

 The case-specific implications of Rowbottom’s four questions for SD are presented in Table. Each case is positioned in the context of its initial objectives and final results. The original questions are interwoven with process steps, insights, and model phases and end up in descriptions of resulting actions, levels of implementation, and identified barriers to implementation. As noted above, all cases had similar beginnings and ends but had different sequencing of intermediary steps as the processes adapted to the needs of the participants. The table shows how each case unfolded over time (vertically) as well as allows for cross-comparison of cases by stage to see similarities and differences between them (horizontally).

**Table:** Understanding the Empirical Material by Use of Rowbottom’s Four Questions

	**Case 1 – Stroke**	**Case 2 – Obstetrics**	**Case 3 – Dementia**	**Case 4 – Paediatrics**	**Case 5 – A&E**
Purpose	Plan for an extension of beds in a new stroke ward and identify qualitative variables to improve health outcomes.	Develop principles for a drop-in system, for post-birth follow-up due to patient dissatisfaction with waiting times.	Developing new shared practises and improved utilization of facilities at a municipal care home for demented.	Map work practices, plan for an absorption of an external sub-unit and understand requirements for larger paediatric facilities.	Prove case for extended A&E premises due to increased patient volumes, peak crowding, and concerns about patient safety.
What is manifest? How is it supposed to work?	Contrary to national guidelines, 45% of stroke patients were placed in other wards, which suggested a need for additional beds.	There was mismatch between staff scheduling and desired arrival times of patients.	Current work practices and facilities were not aligned with latest best practices.	Consequences of increased patient volumes not considered in plans or budget, which could jeopardize patient health.	Examine allocated resources and their utilization, map work practices and understand requirement(s) for larger facilities.
Problems and objectives inventory	All participants were asked to note problems and objectives. Participants were encouraged to ask each other questions in order to understand stated issues, but not go into any discussions. The modeller asked questions to understand the unit, its operations and what was important. At the end of the listing the modeller asked, “Have we really exhausted all problems and issues?” to ensure that everything had been listed. The modeller would then ask a few participants to rearrange all stated issues by theme. The purpose was to signal that participants own the issues, not the modeller.				
Initial model	The initial model, built on stated patient numbers, failed to fill available beds in the present ward.	The initial model, built on current staffing and wished arrivals, showed that unmitigated drop-in would worsen problem due to births seven days a week but receiving patients five days a week.	N/A	The initial model showed a basic patient flow on a daily basis and allowed for studies of seasonal variation.	The initial model showed a basic patient flow varied by time of day and by weekday and with almost constant staffing.
Insights	Results from the group process formed the basis of a causal loop diagram of qualitative factors, with “actual health status” in focus. As to the initial model not filling available beds, participants said “well, you (the modeller) also need to know...”	As the initial model illustrated the effects of unmitigated drop-in, the participants rapidly identified the key issues to solve.	Results from the group process formed of a causal loop diagram of qualitative factors, with “satisfied residents and relatives” in focus.	Results from the group process formed a causal loop diagram of qualitative factors with three foci “patient safety,” “infection protection” and “patient centred care.”	Results from the group process formed a causal loop diagram of qualitative factors with two foci, “patient safety” and throughput time measures.
What is assumed? How do the participants believe it works?	Participants contributed additional data for patient flows that partially used beds intended for stroke patients. They also listed qualitative care factors that they believed contributed to good health outcomes.	Participants and professions views were captured during a tour of the promises and group discussions. Perspectives differed both between and within professions. The modellers asked questions to address their own knowledge gaps.	All participants were united in leaving old work practices behind but concerned about some colleagues unwilling to adopt new practices. The manager assured that he would handle those concerns. Participants openly shared their assumptions of what constitutes good care and how that differed from present ways of working.	The perspectives of the different professions varied since they had distinct hand-overs of patients and rarely worked together. All participants shared deep concerns about patient safety, peak capacity, and future increased patient volumes.	This was a multi-professional group used to working as a team under pressure and rapidly complementing their different perspectives within/between professions.
Revised model/ causal loop diagram	The revised model showed that the ward did not need additional beds. The critical issue was the high flow of patients with non-stroke diagnosis that pushed stroke patients into other wards. A causal loop diagram was built using the qualitative factors.	The revised model allowed for varied staff scheduling and “nudging” of arrivals. A user interface was introduced, so that participants could focus on possible scenarios and outcomes.	The group was facilitated in building a causal loop diagram centring on “patient and relatives’ satisfaction.”	The revised model incorporated the interaction between variations in patient flows and qualitative factors.	The revised model was based on qualitative factors, waiting times for current staffing levels, foreseeable variations in patient flows, time for radiology and waiting time for admission to hospital wards.
Insights	There was a sense of disappointment in the group as the uncovered problem differed from the initially perceived problem.	Participants saw clearer how their work fitted together and how they by minor changes could reduce waiting times.	As participants took part in how they would work in the future they became more and more enthusiastic and engaged.	The revised model led to further discussions like the use of waiting areas, playroom and the separated corridors for out-patients and day-care patients.	The revised model led to discussions about the many factors contributing to crowding, such as accompanying relatives, shortage of examination rooms.
What is extant? How does it actually work?	The conversation changed from increasing number of beds to ensuring vacant beds for incoming stroke patients. Comparisons showed that the hospital’s outcomes were better than national averages, so there was no basis for building a model with qualitative aspects.	The model at this stage was accepted by the participants as it behaved according to their experiences and coped with the random variations in birthing.	Participants were in agreement on what constituted good care and how it differed from present ways of working. Discussion and experimentation with the causal diagram created systemic understanding.	Dynamic simulation showed that concerns were relevant particularly when peak crowding as well as the consequences of future increased patient volumes. Those effects could partially be absorbed by resource sharing between the corridors for out-patients and day-care patients.	Dynamic simulation confirmed issues around foreseeable and random variations in patient flows over time. The model allowed for testing the limits of the system and discussion how to cope with the outliers.
Final model	The final model allowed for testing different kinds of policies to ensure vacant beds for stroke patients and for rapidly moving outpatients that had completed medical treatment.	The final model allowed for testing of minor changes in staffing to adapt to desired arrival times, as well as principles for directing patients to specified half-days and for nudging arrivals to less desired times.	The causal loop diagram was converted into a weighted simulations model using an interface so that the group could test the influence of variables and discuss their priorities.	The final model allowed for testing different kinds of policies to challenge the capacity limits of the unit and to illustrate seasonal and random variations in patient flows.	The final model highlighted mismatches between almost constant staff levels and regular patterns of patient arrivals over a day and over a week, leading to significant queues.
Insights	The group realized that as the unit did not need additional beds, they needed to develop principles for admittance of non-stroke patients to ensure beds for their target patients. The final model allowed for testing suggested principles.	The major “aha” moments were understanding the mismatch between births seven days a week but receiving patients five days a week and realizing that patients could be nudged to arrival times instead of being scheduled.	Discussions around the causal diagram led to shared understanding of what practices had strong effect and which factors to prioritize, when moving to knowledge-based and person-centred care.	The major "aha" moment of the group came when testing the limits of the system – peak crowding. The group said that this was exactly how it had been when respiratory syncytial virus peaked. At that point, the group felt that the model was validated and spent quite some time experimenting with it.	The major "aha" moment of the group came when they doubled the average patient inflow. The reaction was “this is what it looks like when it is stasis,” at least once a week. Action proposals were developed based on objectives and model outcomes.
What is requisite? How could it work?	A small number of policies to reserve beds for stroke patients were discussed and tested in the model. The group noted that the main obstacle was gaining acceptance from management and other units for withholding beds.	A final proposal for drop-in was developed. The proposal was tested and fully implemented after minor changes. Both patients and staff were highly satisfied.	Staff and management were very satisfied that they had a shared map and prioritized pathway from the present ways of work to the desired.	Participants saw that the results of the simulation could support their position as it clearly showed the effects of peak crowding and the satellite closure.	The results confirmed perceptions around cramped facilities. Patients flows peaked every afternoon/evening while staffing was constant, which led to foreseeable queues. Patients also needed to be admitted to wards faster.
Degree of implementation*	Conceptualised	Implemented	Conceptualised/ Implemented	Conceptualised	Conceptualised
Barriers to implementation	The simulations showed that no additional facilities were needed.	N/A	Results were integrated into the ongoing organisational transformation and implemented later.	The department was a satellite of paediatrics at a regional hospital but localized at a rural hospital. Participants expressed that they felt unseen by both and concerned that the need for investment in larger facilities would not be heeded.	Considerable investment needed, to be handled in planning and budget process.

Abbreviations: A&E, Accident and Emergencies; N/A, not available. * Degree of implementation based on Brailsford^24^: Suggested, theoretically proposed by the modellers; Conceptualised, discussed with a client organisation; Implemented, actually used in practice.

 The initial problem statement was dismissed in Case 1 and an uncertainty among members of the group arose. This could not be addressed effectively as neither the manager nor the medically responsible physician were present during meetings. As a consequence, discussions focused on how to stem the inflow of non-stroke patients. In Case 2, the work in the group led to the development of potential solutions and a test in reality, after which some modifications were made in preparation for the final deployment of the solution in which the modellers were asked to take part in. In Case 3, the group process and the causal loop diagram and the simulations laid the foundation for continued action planning and contributed, in part, to the subsequent reorganisation of work. In both Cases 4 and 5, the key issue was cramped facilities, which required a more extensive investigation and had to be addressed in an investment budget process outside the scope of the initial objectives. The models indicated that a possible short-term solution might be found, in Case 4, by spreading patients evenly across the available space and, in Case 5, by a better matching of staff levels to foreseeable patterns of patient arrival times.

In all five cases, the starting points were based on issues or problems where stated work principles did not work as intended. All cases went through an AR-based inventory of problems and objectives. Initial fact-finding was carried out and an initial SD model with narrow scope was built and/or a causal loop diagram was created based on the problem statements. This led to a first overview of the interconnectedness of the issues at hand. In two of five cases, the model did not replicate reality as perceived by the participants prior to this phase. In the next phase, the participants brought in their assumptions in the form of their respective knowledge and experience about their work situation and what was missing in the models. The models were finalized and became extant as they replicated the realities perceived by the participants. In all improvement cases there were forms of aha-moments when the simulations clearly pinpointed problematic situations and their causes. Once the root issues and causes were present in the model, then insights were shared and perceived as clear to all, the participants moved to suggesting solutions. Proposals were tested *in silico* in all cases, discarding those that were deemed to have none or little effect finally arriving at requisite proposals of solutions.

####  The Facilitating and Modelling Perspectives

Almost all meeting time was spent in facilitated group discussions and, in all cases, most of the modelling work was conducted between meetings (Supplementary file 4). There was engagement, interaction, and exploratory learning activities throughout each case. Meetings began by the facilitator asking the participants for reflections since the last meeting. Typically, the participants reported that they, based on thoughts after the previous meeting, had seen their work in the light of a somewhat new perspective. Likewise, they were also asked to report on their reflections at the end of each meeting, leading up to ideas about what to observe before the next meeting. Facilitation ensured that the voice of each group member, and profession, was heard at all meetings in that the participants were repeatedly being encouraged to talk in turn. Although it was not made explicit to the participants, AR and SD were combined throughout the entire processes. Initially, the participants were hardly introduced to SD because the focus was on a group process that contributed to the uncovering of problems and to the development of a shared view on the current state of affairs (cf. Rowbottom: manifest and assumed). However, already during the initial steps, the outline of a model was considered by the facilitator/modeller – including which variables to include and what data that were available.

As an overall work principle, the process moves from left to right in Figure 1. As each project progressed, the model and simulations became explicit and took more of the meeting time. During the experimental phase and final workshop, the participants were fully absorbed by the model and simulations, but the ongoing group process was still considered central and was focused by facilitation in line with AR principles. In none of the cases did the participants have to learn the basics of model building or SD terminology. However, they understood sufficiently to see the simulation results as credible and useful.

####  Group Composition

 Group members of each respective case shared the same workplace (Cases 2-5), but four of twelve participants in Case 1 came from an external department (facilities management) and predominantly took part as observers. In all workplaces, patients were handed over from one profession to the next, a process that required coordination between staff members rather than collaboration as the professions had different roles and accountabilities. However, in Case 5, the staff interacted more extensively to the point of collaboration.

The group sizes in Cases 2-5 were close to the ideal 6-7 participants as shown by Sjölund.^36^ Those groups also had Coalesced Authority, Power, and Influence (CAPI) required for decision-making.^37^ They had high levels of engagement and rapidly moved forward through the process. However, the group in Case 1 was large, had non-active participants, and did not have CAPI as neither the manager nor the medically responsible physician was present. This resulted in the process being impeded.

## Discussion

The aim of this study was to explore and identify methodological aspects of how the integration of SD into AR supported multi-professional groups in healthcare to discover workable solutions to work-related challenges. We found that all of the cases studied began with an extensive AR-inspired inventory of problems/objectives and ended with an SD-facilitated experimental phase where mutually agreed upon solutions were tested *in silico*. The sequence of intermediate steps and activities was case specific, but the majority of cases included the development of causal loop diagrams and graphical user interfaces to the respective SD model. The time was predominantly divided between discussions/reflections during meetings and modeler/modelling work between meetings. There was an overall pattern of continuous oscillation between divergence and convergence, but in two major phases, as each group moved towards a mutually developed point of reference, a consolidated case-specific *multi-professional knowledge repository. *Work principles ensured that the voice of each participant was heard, inspired engagement, interaction, and exploratory mutual learning activities among both participants and modeller/facilitator.

###  Divergent and Convergent Phases

Guilford^38^ defines divergent thinking as creatively going off in different directions and leading to a diversity of answers, where more than one may be acceptable, whereas convergent thinking narrows down the number of answers. We found that the studied cases went through two major divergent/convergent phases (Figure 4). The first phase focused on generating a comprehensive listing of potential problems connected with the issues at hand. This led to participants understanding problems as seen by others and how all issues fitted together. Divergent phases need to be exhaustive in order to avoid premature convergence,^39^ which otherwise could lead to sub-optimal solutions or low acceptance among participants or in the client organization. Fact-finding was carried out as needed by the identified issues. During the second phase, ideas and solutions were brainstormed. All suggestions could immediately be tested *in silico* and evaluated as potential solutions. Finally, the investigated mixed-methods approach led to acceptance as each participant saw how their work fitted in with others and gave a more comprehensive understanding of their system. These findings are in line with results presented by Parnes.^40^ He summarizes problem solving in five stages, each divergent and convergent: (1) fact-finding, (2) problem-finding, (3) idea-finding, (4) solution-finding, and (5) acceptance finding, also emphasizing the aspects of creative divergent brainstorming activities and convergent focusing to this problem-solving model.

###  Multi-professional Knowledge Repository

In this study, we found that the participants brought their reality into the process, used their insights from the meetings to observe and re-assess their realities between meetings, and, in turn, brought their reflections into the next meeting. This “feedback” improved both the insights of the group altogether as well as quality of the simulator under development, and it contributed to the identification of actionable solutions that were tested in the virtual reality of the simulation, as suggested by Scholl.^26^ At the same time, the work principles applied assured that the overall group process was nudged towards satisfactory outcomes. The simulations had a catalytic role to play in the overall process and represented an agreed-upon comprehensive repository of knowledge. This study extends the results of an earlier in-depth study of Case 2,^28^ which concluded that the participants were not stakeholders in the sense that they held firmly competing opinions and felt compelled to persuade each other.^10^ Instead, it was a co-creative process, where participants explored how each person’s set of knowledge was aligned with that of the others and did so in an experiential^41^ and reciprocal^26^ learning process that resulted in the accumulation of the here reused concept of a *multi-professional knowledge repository*. They thus became shapeholders^42^ rather than stakeholders.

###  Integrating 

####  Studying the Interaction of Integrating System

In contrast to scripted GMB, we found that the process of integrating SD into AR in a more fundamental way addressed the concerns of participants and laid the foundation for actualisation. We found that Scholl’s^26^ suggestion of reciprocal learning, ie, convergence, between reality and the simulation model contributed to the investigated work process. Both AR and SD influence how people think. SD provided a form of gaming or role-playing platform where experiential learning took place. One of our key observations is that a group process rooted in AR leads to shared mindsets and to the realization that it is the mindsets, rather than a top-down implementation project in itself, that increase chances for actual change. AR processes are, in a sense, self-implementing.

We are in agreement with Mingers and Gill^20^ that using several methods indeed provided a richer multi-facetted representation of the problems and potential solutions than what the application of either of the two methods in isolation would have provided. Using the mixed-method approach described here, we found that the groups tested a multitude of potential solutions in a very short time, discarded what did not seem to work, and converged on robust proposals that satisfied objectives and were accepted by all. A “pure” AR process usually has a considerably higher focus on reflexivity, group development, and iterative testing of actions in reality.^43^ A “pure” SD process has a focus on the building of a fully formed and rigorous model with a strong emphasis on feedback loops and non-linear interaction effects, and the complex dynamics that result from these.^44^ For GMB, where AR is integrated into SD, Oyo et al^45^ further underlines the importance of rigour in the modelling process but also discusses Scholl’s proposal of integrating SD into AR. To the best of our understanding, few studies have otherwise reported on this particular approach. Searches in Scopus, and in conference proceedings of the International System Dynamics Society in December 2020, using different combinations of the search terms “SD and AR” for papers detailing the integration of SD into AR resulted in three papers including Scholl. Holmström et al^28^ reports on an in-depth study of one of the cases in this paper, integrating SD into AR, and Walker and Haslett^27^ provides a case description of the outcomes of an AR process using SD. The latter, however, does not describe the characteristics of the combined process as such making it difficult to compare their data to the findings reported here. Furthermore, Rosmulder et al^29^ reported on AR used with another OR method in healthcare (discrete event simulation) and, similarly as suggested by our study, conclude that integrating simulations with an AR process is a promising combination to promote action in reality.

####  Facilitation

Group process facilitation is a vital part of the AR process. Vennix^46^ also notes that group facilitation is one of the most crucial elements in effective GMB in SD, when facing complex and “messy” problems as well as divergent stakeholders. Facilitators are usually transparent about how they are to proceed, but not necessarily explicit about the theory and practise of their work, as this could shift the focus from the work of the group to the facilitator. Straus^47^ states that facilitators rely on interventions or preventions. The former is applied to keep a meeting on track when obstacles occur, and the latter includes actions taken before or during meetings to eliminate potential barriers to success. Straus^47^ claims that the most skilful facilitators rely on preventions. As shown in Figure 2, case flows were pragmatically adapted to the facilitator’s perception of “where the group was.” This made his work invisible, so that participants appreciated their own work rather than that of the facilitator. All the cases studied began by facilitating the problem identification (including a process of clarification), where the purpose was to uncover all problems of concern, listen to and take note of the problems described without judgement and, for the group, to gain trust and acceptance in the facilitator and the process. This laid the ground for a way forward whereby participants would feel safe when challenging the modelling process as well as when challenging the simulation results and the subsequent interpretations. The facilitator also needs to be trusted in order to be a to be a “comforter” and to – temporarily – contain the anxieties stirred up by the uncertainty, ambiguity, and potential conflicts in an emergent process.^48^ The nature of this facilitation process caused the participants to take ownership in the solutions and to take responsibility for their actualization.

####  Strengths and Weaknesses

The presented re-analyses relied on a large body of contemporaneous notes about the improvement cases that were revisited and interpreted during the research process. The qualitative abductive approach, using initial constructs as a basis for analysis and preparation of displays that were discussed and reinterpreted in a multidisciplinary setting, iteratively lead up to new insights and clarifications that caused the perspectives of the research group to meld. Eisenhardt^31^ describes how theory evolves in constant iteration, backwards and forwards between steps, and suggests that *a priori *constructs can shape the initial design of research creating new theory-building from multiple cases. We consider this as one of the strengths of our study. Any retrospective analysis may, however, be subject to recall bias on the part of assessed data and should be considered a possible limitation. More extensive and clearly structured notes and contemporaneous reflections in this study could have contributed to a deeper analysis. However, the support and rigorous questioning from the multidisciplinary group of researchers and the objective handling of the material may well have compensated for this. Had the research documented in this study been planned in a prospective setting, the experiences of the participants could have been recorded through independent observations and interviews. On the other hand, the act of recording, observing, transcribing, and measuring a process may cause an observer effect, affecting the behaviour of the participants and potentially influencing the process.^49^

As AR is situational, the outcome when integrating SD into AR is likely to be affected by this feature, ie, if the process is repeated it would neither be identical, nor would it produce the exact same results.^26^ The presented findings should, therefore, be taken as guidance to others to adapt to the situations that they encounter. It would also be helpful if the act of integrating SD or other OR methods into AR processes were described by others to enable us to understand how the investigated approach performs in other areas of healthcare as well as in other contexts.

## Conclusion

 Using a mixed-methods approach, where SD is integrated into AR, we found that there is potential to achieve outcomes that are more useful, comprehensive, and robust for the client organization than when applying either approach in isolation. From the investigated improvement cases in healthcare, we found that AR contributed to a high level of engagement among the participants and to the building of confidence in and ownership of the results. SD provided a coherent and consistent systems overview of the complex and complicated structure of each case that we studied, offered causal rigor, and provided ample opportunities for reality checks. The overall AR/SD process came together in two major divergent and convergent phases that stimulated creativity as well as led to a timely congregation of mental models. The process also led to shared points of reference for the problem as such, helped to identify robust solutions, and increased understanding of systemic effects resulting from putting suggested solutions in action. The results were calibrated to local needs and circumstances and created a higher likelihood of sustained actualisation compared to what results from a traditional top-down implementation typically would have done.

## Acknowledgements

 The original case work was funded as consultancy assignments carried out by the first author. Permission was given to use any results academically but anonymized. The first author is retired and has worked on the manuscript in his own time. CO is the main supervisor of the first author in his PhD studies and is partially funded by Swedish Research Council (2017-01753). The other authors have contributed as supervisors and part of their organizational roles.

An early version of the abstract by the same authors, containing aim, materials and methods was presented as work-in-progress at the 39th International Conference of the System Dynamics Society on July 21, 2020, virtually in Bergen, Norway, with the title “Insights gained from a reanalysis of five model-facilitated change processes in healthcare based on action research approaches.” The abstract can be found at: https://proceedings.systemdynamics.org/2020/abstracts/1192.html.

 We would also like to thank our anonymous reviewers for helpful comments to improve previous versions of this manuscript.

## Ethical issues

 No ethical approval was needed for this study since it builds on already collected case data without personal details about participants.

## Competing interests

 Authors declare that they have no competing interests.

## Authors’ contributions

 Conception and design: PH, PD, FB, and CO. Acquisition of data: PH. Analysis and interpretation of data: PH, PD, FB, and CO. Drafting of the manuscript: PH. Critical revision of the manuscript for important intellectual content: PD, FB, CO, and TBE. Supervision: PD, FB, CO, and TBE.

## Supplementary files


Supplementary file 1. Case Descriptions. Detailed descriptions of the five studied cases.

Supplementary file 2. Analysis of Analytical Iterations. Descriptions of the major iterations of qualitative analysis.

Supplementary file 3. Rowbottom’s Four Socio-Analytical Questions. Rowbottom’s four socio-analytical questions and their implications for SD when working with groups to solve problems in work systems.

Supplementary file 4. Time Spent on Facilitation or Modelling. Estimation of share of time spent on facilitation or modelling by step in the generalized workflow where a combination of AR and SD was used to identify actionable solutions to problems in healthcare.
