# Supplementary material for: Insights Gained From a Re-analysis of Five Improvement Cases in Healthcare Integrating System Dynamics Into Action Research
Source: Int J Health Policy Manag. 2022 Feb 26;11(11):2707–18. doi: 10.34172/ijhpm.2022.5693 (PMC9818115; doi:10.34172/ijhpm.2022.5693)
Supplement: Supplementary file 1 — Case Descriptions. Detailed descriptions of the five studied cases. [file ijhpm-11-2707-s001.pdf]

Article title: Insights Gained From a Re-analysis of Five Improvement Cases in Healthcare Integrating System Dynamics into Action Research

Journal name: International Journal of Health Policy and Management (IJHPM)

Authors' information: Paul Holmström[1,2]*, Thomas Björk-Eriksson[2,3], Pål Davidsen[4], Fredrik Bååthe[5,6,7,8], Caroline Olsson[1,2]

[1]Department of Clinical Radiation Sciences, Institute of Clinical Sciences, Sahlgrenska Academy, Gothenburg University, Gothenburg, Sweden.

[2]Regional Cancer Centre West, Gothenburg, Sweden.

[3]Department of Oncology, Institute of Clinical Sciences, Sahlgrenska Academy, Gothenburg University, Gothenburg, Sweden.

[4]Department of Geography, University of Bergen, Bergen, Norway

[5]LEFO – Institute for Studies of the Medical Profession, Oslo, Norway.

[6]Institute of Stress Medicine, Gothenburg, Sweden.

[7]Sahlgrenska University Hospital, Gothenburg, Sweden.

[8]Institute of Health and Care Sciences, Sahlgrenska Academy, Gothenburg University, Gothenburg, Sweden.

(*Corresponding author: Email: paul@holmstrom.se)

Supplementary file 1. Case Descriptions

Case 1 – stroke ward

Background

The project was initiated as part of a research program studying the usefulness of simulation modeling as a preplanning tool in the design of new health care environments. Here a stroke ward was studied. According to national guidelines stroke patients should be treated at a dedicated ward. At the hospital in question 45% of all stroke patients were placed in other wards as the stroke ward was working above capacity. The purpose of the project was to lay the ground for a care planning process and health care environment at a new larger ward.

Participants:

Stroke nurse (contact with rehab, primary care and national stroke registry), ward manager (stroke, neurology and diabetes), manager neurology open clinic, care development medical clinic, auxiliary nurse stroke, head physiotherapist medical clinic, manager medical clinic (only attended the initial meeting), physician neurologist (never attended), facility manager, health planner county council (liaison between care and facility management), planner at the facility management company (former nurse), architect, also active in the research project and an architect

In addition to this a research project leader and a modeller participated. The impression of the modeller was that the group was composed so as to be representative of all the involved stakeholders. The attendance was high, there usually were 13 people present. The participants involved in architecture and facilities mainly took part as observers.

After the first meeting, each meeting began with reflections since last. All meetings ended with reflections and summarizing was supposed to be done before the next meeting. However, as time was short and there were many participants, all voices were not always heard.

Start stage

1 Problems and objectives inventory

At the initial meeting the care unit manager stated that the need for new and adequate premises should under no circumstances be questioned and described the main problems:

- Too few beds (8-10 at the stroke unit), led to patients being placed on other wards. To be prepared for the variation in the inflow of stroke patients, the ward needed to have a capacity utilization of 85%, which then was 102%.
- The number of stroke patients would increase by 30% over a 10-year period
- The facilities were not entirely adapted to the needs of the stroke unit
- The staff (physician, stroke nurse, occupational therapist, and physiotherapist) had to move between other wards where stroke patients were placed, which takes time.

At the first meeting the modeller used brainstorming techniques to elicit care-related parameters that influenced the health outcomes of the patients. Participants were first given time to jot down their thoughts, then to share them, as the modeller wrote all points on a whiteboard, clustering them. Time was given to reflect and ensure that all had their say. It was clear that there were somewhat different aims of the stakeholders in the group that needed to be reconciled.

At the second meeting another brain-storming round was carried out to elicit parameters related to the premises that effected health outcomes. By then the participants had been introduced to an initial causal loop diagram based on the care-related parameters which led to discussion of causality already during the elicitation and parameters were clustered.

Intermediary stages

2 Qualitative causal loop diagrams

Prior to the second meeting the modeller drew a causal loop diagram of the care-related parameters, discussed it with the project leader and made revisions (Figure 1). The modeller prepared a series of ten PowerPoint slides gradually building up the causal loop diagram, which was presented at the second meeting. Many of the participants were actively involved in discussions, clarifying terms and loops. After the presentation the modeller moved to a software application for causal loop diagrams and made changes together with the group. The participants were not inclined to simplify the diagram; the tendency was instead to add more detail.

Again, the modeller collaborated with the project leader to draw a formal causal loop diagram of the parameters related to the premises, which was presented and discussed at the third meeting (Figure 2). There was little discussion at this time. The participants were then showed the two sets of causal loop diagrams together and noted that they would connect at several points, but that we would not do that as it would become too messy to overview.

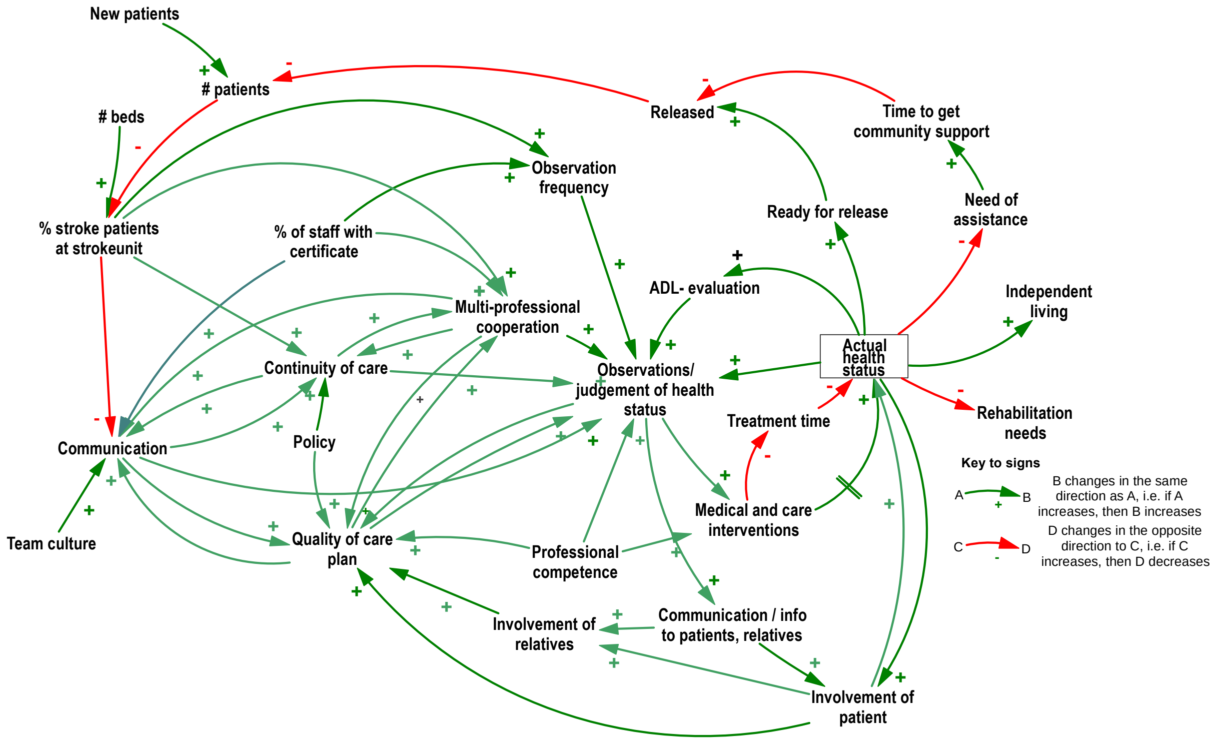

Figure 1 Case 1 - Causal loop diagram - care related parameters for stroke patients in the acute phase

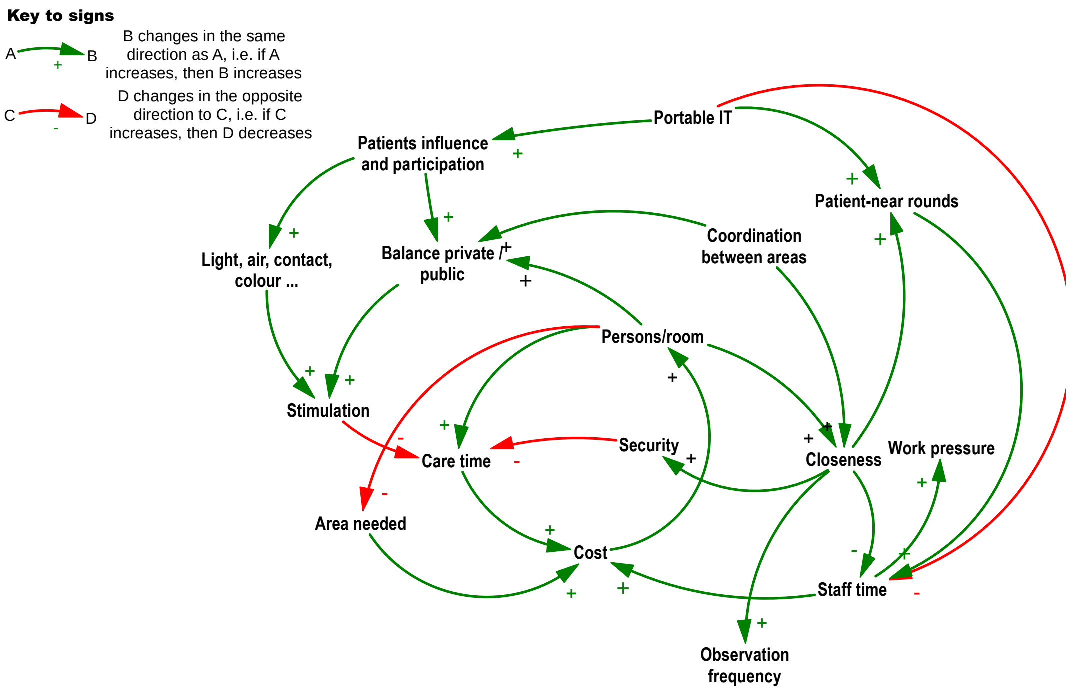

Figure 2 Case 1 - Causal loop diagram - room related parameters caring for stroke patients in the acute phase

Both causal loop diagrams were considered as tentative, to be revised after a literature review identifying key factors influencing patient outcomes. The intention was to, in a second stage, go beyond the initial expansive elicitation of factors to a condensed diagram with named loops. As noted in section 6 "Problem visualization in model" below, this did not happen. As they stand, both diagrams reflect the complexity of caring for stroke patients in the acute phase. Time from onset of stroke to initial treatment was noted as highly critical, a timely and correct diagnosis must be made. Several medical professions are involved in patient care and there is a need for them to communicate and coordinate closely with each other. According to national guidelines, stroke patients should be treated in dedicated wards, but at the studied hospital only 55% were so. Figure 1 shows that clusters of factors such as ratio of patients assigned to the stroke unit, professional competence, coordination, and planning, have effects on patient's health outcomes, rehabilitation needs, and ability to live independently.

3 Initial model

Before the third meeting the modeller prepared a simple model with a patient flow, disease trajectory and staff and room capacity. Based on a random inflow of 462 stroke patients no more than seven of the12 available beds were used. After the initial reflections at the third meeting the model was shown, and participants asked what had the modeller misunderstood. The ward manager explained that the ward takes in another 222 patients per year with suspected stroke, that were not included in the statistics as they are discharged with another diagnosis. The modeller amended the simulation, all beds were still not utilized. The ward manager then explained that the ward in total has 22 beds of which 10 are for acute kidney issues, fairly often that patient group took up 12 beds, leaving only 10 for stroke. Also, the emergency department often called and placed patients with other diagnoses on the ward as other wards were full. The hospital had 98% bed utilization making it difficult to access beds.

The modeller continued developing the simulation model before the group, explaining each step in the process. Three outflows were added to rehab at the hospital, nursing home or independent living with support. Each outflow with a stock of medically treated, waiting for placement. The participants were asked to estimate how much of the care time was actual treatment and waiting time.

4 Fact-finding

After the third meeting the modeller sent the project leader a list of data needed to populate the model. The list contained both patient-flow related data and health outcome comparisons. It became clear that some data did not exist, so the model needed to be adapted to what was available, initially focusing on patient flows and later adding qualitative data and health outcomes.

5 Patient flow diagram

The modeller prepared a Sankey-diagram over the patient flows as an intermediary before building a detailed SD stock-and flow diagram (Figure 3). As the diagram reflects patients flows as the group understand them, it serves as a point of discussion and confirmation before moving into detailed modelling, which can be abstract for participants.

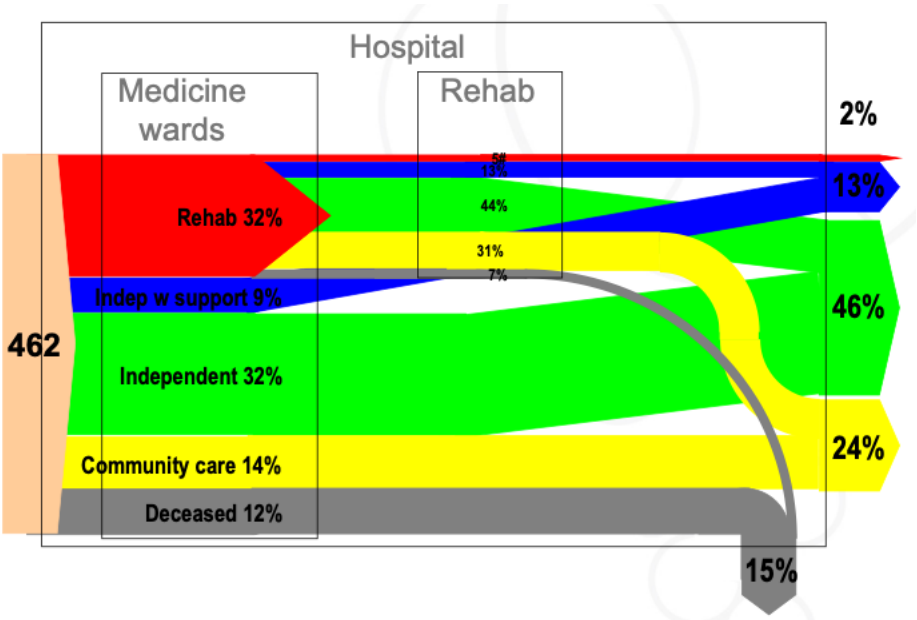

Figure 3 Case 1 – Patient flow diagram

6 Problem visualization in model

The project leader and the modeller decided to build two models. The primary focus was to continue the quantitative modelling to follow the patient flow diagram while also taking into consideration the effects of using beds for kidney patients and other patients from the emergency department. The model was validated in the sense that it could be initialized with data so that 45% of stroke patients were assigned other wards, and to allow testing of all policies that had been mentioned to lower that number.

The second model was to be qualitative, building on national and international data applied to the parameters in the early causal loop diagrams. However, as the hospital had better health outcomes than national averages despite 45% of patients being treated in other wards it was difficult to see why such a model should be built. The project leader and the modeller decided instead to test a scoping questionnaire with the participants where they could roughly estimate health outcomes of specific actions. As the participants did not consider themselves able to make such judgements, the group instead were asked to prioritize the different actions.

7 User interface

A user interface for the quantitative model was built with the following variables:

- Number of stroke beds in a range 12-20
- Reserved beds, 0-5
- Ratio of suspected stroke, but other final diagnosis
- Switch allowing kidney patients to take 2 beds or not
- Switch allowing patients with other diagnoses from ED or not

And graphs

- Accumulated share of stroke patients placed at wards
- Bed utilization at wards
- Average # stroke patients
- Average medically treated, waiting for discharge
- Average # suspected, but non-stroke
- Average # others

The user interface had a translative function between the reality of the participants and the actual simulation model. The participants needed a basic understanding of the system dynamics model so that they

could decide which variables they want to experiment with in the user interface and which outputs they want to see in graphs or tables to see the effects of their experimental decisions. Having a user interface directly involved the participants in asking "what-if" questions once the modellers have demonstrated some experiments.

A user interface was also built for the qualitative model. As data still was being gathered, the initial assumptions were shown in tables so that they were transparent and could be changed. The model also had five sliders for the key variables and two graphs showing three health outcome variables.

End stages

8 Scenarios, model simulation and experiments / Workshop

Based on suggested policies and actions the modeller prepared a series of scenarios running the quantitative model at the fifth and final meeting. Each scenario was discussed in detail. The main insights were

- As long as the hospital had near 100% utilization of bed capacity, increasing the number of beds at the stroke ward would have little effect as they would rapidly be filled with other patients arriving at higher rates than stroke patients
- Switching off the inflow of other patients and not allowing kidney patients take beds led to sufficient bed capacity for stroke patients even allowing for caring for patients with suspected stroke
- Attempting to reserve 1-2 beds for future stroke patients and not taking in other patients had a significant effect of the ratio of patients treated in the stroke ward.

Comparisons with national qualitative data were presented and noted as inconclusive as the hospital had better outcomes than national averages.

9 Conclusions

The project undertaken had financing for five meetings. There were difficulties getting data timed with the meetings, but the meeting schedule was adhered to anyway. Substantial work was provided pro bono afterwards using data when it became available and improving the quantitative model. In addition, the project leader and modeller met five times over three months. They were in agreement that the qualitative model was a disappointment, since the medical evidence did not support a dynamic model and now that patient data was available, the data showed that the ward was better than the average well-organized ward.

The main conclusion of the project was that the initial assumption that the ward needed additional beds were incorrect and that the manager needed support in reserving bed capacity for future stroke patients.

Case 2 - Obstetrics

Background

3-5 days after a birth, mother and child are followed up. One important purpose is to identify any problems or complications and perform a phenylketonuria test for genetic disorders on the baby. The test is time-critical as it needs to be carried out 3-5 days after the birth. As stays in the post-natal wards have been shortened, the mother and child come back to the department for the follow-up. The consultation is scheduled when they leave post-natal care.

The consultations were planned to take 30 minutes. Weight was checked, if reduced, then breastfeeding was discussed, and advice given. The midwife looked for any indications of complications, ill health etc. If so, tests were taken. However, there was significant variability in used time. The follow-up for first-birthers could take longer. For those coming with their second or later child, little follow-up was usually needed.

There were a number of problems such as having sufficient time slots to schedule within the given time frame and people not arriving at the scheduled time, disrupting planning and causing stress among the staff. A different procedure had been discussed, based on drop-in principles. The queue issue mainly depended on variation in birthing numbers, but also problems in adhering to reserved times. A simple survey had been carried out to find out when patients wanted to arrive during the day. As there was a mismatch between desired arrivals and staff scheduling the project at hand was initiated.

Participants

2 midwives, 1 assistant nurse and 2 managers. In addition to this, two modellers and a representative from the quality department of the hospital.

After the first meeting each meeting began with reflections since last. All meetings ended with reflections and summarizing who was supposed to do what before the next meeting.

Start stage

1 Problems and objectives inventory

The intention was that the participants should tell everything about issues and problems that they perceived. We went around the table multiple times, each participant adding to a list on the whiteboard. The modeller wrote all points without rephrasing, noting them close to similar items after checking that it was so and actively asking questions to understand the issue at hand, and so that participants would feel listened to and be reassured that somebody wanted to assist them in solving the issues and were credible in doing this. After a few rounds some participants did not have anything to add, but they were queried again in the next round in case they had thought of something to add and to ensure that all problems and issues were covered. When the end of the listing seemed to be reached the modeller asked “Have we really exhausted all problems and issues? At this point it is important that everything is on the table! Let´s take a short break and we will revisit the list when we are back.”

The modeller needs to quickly sense the feeling in the group and direct the conversation; accordingly, are they resigned over all the problems or optimistic about finding solutions?

A small system dynamics model was shown to illustrate how patient flows can be simulated, also illustrating the effects of significant random variations on queues.

The modellers expressed a need to fact-finding visit the premises to see the actual patient flows, gather data and see scheduling systems etc.

Intermediary stages

2 Fact-finding

The fact-finding tour was led by participants of the group, the modellers were there to hear their story about their work situation and get a sense of patient flows, the work carried out and roles of midwives and assistant nurses. This was not a directly facilitated meeting. The group process was to ensure that the participants “owned” the meeting. Midwives told how they sat in consulting rooms meeting patients and opened doors to the crowded waiting room. Assistant nurses described how they oversaw and buffered patient flows by carrying out their part of the visit before or after the midwife. After the tour in the premises the modellers asked to see the system for planning and scheduling meetings and asked for data such as birthing statistics, patient flows and staff scheduling.

3 Initial model

Prior to the initial model two graphs were introduced and discussed. The first graph showed the mismatch between desired arrivals and staff scheduling on an average day. This did not lead to much discussion as all were aware of the issue. The second graph showed needed staff capacity per weekday base on the time constraints of the tests and the fact that births occur daily, and consultations are on weekdays led to higher demand on Mondays and Fridays since the unit was closed over weekends. This surfaced a problem that participants were not aware of and led to extensive discussions as there in practise were fewer time slots available Mondays and Fridays. After discussions clarifying the issues and suggesting solutions the modellers demonstrated a small initial model with the intent of showing system dynamics in action as well as illustrating two facts that needed to be addressed with the work group: [1] babies are born all weekdays, but the consultations were only done weekdays. To hold the time frame of 3-5 days, more capacity was needed Mondays and Fridays; [2] There was no match between desired arrival times during a day and staff scheduling. As the purpose of a drop-in system was to take care of all arrivals the model showed as if patients stayed overnight or over the weekend in the waiting room. This helped to create a sense of urgency and stimulated another round of discussions.

4 User interface

The modellers showed and described the underlying simulation model, but also built a simple user interface so that the group could focus on variables that they could influence and their effects on results. As the model iteratively was extended, so was the user interface. The interface contained inputs such as hourly patient arrivals and scheduled staff levels that reflected decisions that the group could take and graphical outputs such as number of patients in the waiting room and waiting times.

Having a user interface directly involved the participants in asking “what-if” questions once the modellers had shown some experiments. The rapid direct question-response stimulated investigation and led to

creativity and the surfacing of many inquiries. It also led to intense discussions between participants about actions rather than discussions between participants and modellers about model refinement.

5 Problem visualization in model

The initial model visualized key issues and led to discussions about possible solutions that a model needed to reflect. Drop-in based on patients desired arrivals and constant staffing levels during day and week, leading to excessive queues patients desired arrivals, constant staffing levels during day and higher Mondays and Fridays, showing that patient arrivals and staffing during days needs to be addressed.

Partial results became insights for the client perspectives on improvement measures and for the modellers perspectives some clarifications on scheduling and work principles, additional data collection as to how patient flows are directed during the day.

As participants understood the user interface and asked relevant questions both the interface and model underwent rapid development to be able to answer questions and test "what-if"-scenarios.

6 Action proposals

The user interface stimulated inquiry and the development of action proposals that were further developed and refined during the scenarios, model simulation and experiments phase. The participants were highly active, and the role of the modeller was to stimulate discussion and support it by rapidly testing inquiries. Many actions were proposed and a set of principles for drop-in were developed based on

- patients desired arrivals and current staffing levels during day and week, leading to excessive queues
- patient desired arrivals, current staffing levels during day and capacity added Mondays and Fridays, leading to excessive queues
- nudging patient arrivals matched to current staffing levels during day and capacity added Mondays and Fridays, showing that a solution may be possible
- staff scheduled according to patient arrival wishes, such varied staffing not possible in reality
- additional staff before lunch break and at end of day
- patient arrivals matched to a number of different staffing levels during day and capacity added Mondays and Fridays.

Final stages

7 Scenarios, model simulation and experiments

As action proposals became firmer, they were tested by experimenting with scenarios, based on concerns raised by the participants

- arrivals with and without random variability, testing fixed and variable additional staffing.
- patient arrivals matched to current staffing levels during day and capacity added Mondays and Fridays, with shorter consultation times all the time or when queue. The latter showed little effect
- seasonal variation +/- 15%

Again, participants were facilitated to experiment and discuss using the modellers as intermediaries between themselves and the user interface.

If drop-in was implemented with current staffing and patient desired arrivals, then the situation rapidly would become untenable. What-if analyses, increased staffing, guiding patient to specific days/half-days/times – including realistic as well as utopian scenarios, showed that solutions were possible.

Constructive suggestions came out during the meeting and were noted on flipcharts. The modellers contributed with practical experiences with some suggestions and refinement of some ideas.

8 Workshop

At this stage a set of action proposals had coalesced. The purpose of the workshop was to test the set of proposals under the different scenarios:

- patient arrivals matched to current staffing levels during day and capacity added Mondays and Fridays, with and without random variability, showing that a solution may be possible
- patient arrivals matched to a number of different staffing levels during day and capacity added Mondays and Fridays

- arrivals with and without random variability, testing fixed and variable additional staffing.

The meeting was led by the modellers, summarizing the proposals, and running them under the different scenarios and stimulating discussion so that participants could finalize their proposed actions.

The group worked through a list of proposed changes, ran them against different scenarios of patient arrivals and selected which proposals to implement.

9 Conclusions / action decisions

The group planned and prepared for a meeting with all staff to be led by the managers, proposing a pilot project based on the final proposals. The modellers participated by demonstrating the selected scenarios to support the proposed changes. The suggested actions were accepted and tested for a month after which minor changes were made before the final implementation, which was evaluated showing good results.

Case 3 – Dementia care

Background

The overall purpose of the project was to study the usability of System Dynamics to support pre-planning architectural processes. The brief was to use System Dynamics modelling to explore mainly qualitative, but also quantitative aspects of work processes to engage staff into considering development of work processes before specifying future needs of premises.

Here a dementia care unit in a municipality in a rural area was studied. The council elderly care manager was lead on the client side. They were in a major transition from dementia care as ”storage units” to revitalizing environments and care. Prior to the intervention many staff members had taken academic credits in dementia care at the nearest university. They were about to change their organisation and move into the pre-planning phase for the nursing home. The purpose of the manager was to move the group from discussions around traditional building programming and engage them in exploratory discussions of their work. A nearby municipality was seen as a warning example, where they had invested substantially in a new building, but not changed the work methods or care itself.

Participants

Seven to nine persons took part, including the council elderly care manager, assistant nurses, and care assistants. In addition to this a research project leader, an architect, and a modeller

After the first meeting each meeting began with reflections since last. All meetings ended with reflections and summarizing who was supposed to do what before the next meeting.

Start stage

1 Problems and objectives inventory

The first meeting began with mutual introductions. The researchers and the staff at the dementia care unit shared their purposes and desired outcomes. Their major objective was “We will build the best dementia care in Sweden”. Donabedian’s framework for examining health services and evaluating quality of health care was introduced and the participants first worked individually and then in pairs to discussed and described the desired outcome, processes, and structure. They wrote on large adhesive notes and presented and discussed their results. A subset of the group clustered the notes according to themes and the participants were allotted a small number of coloured adhesive dots to stick to the notes to indicate their personal priorities.

The inventory was continued at the second meeting after that the chairman of the council committee for social services presented challenges and objectives for the care of the elderly from the political perspective. The meeting moved on to organise and elaborate on the output from the first meeting, first working in pairs, then sharing with all:

- What are our *objectives*? (pink notes)
- What activities are required to achieve the objectives, i.e., *process*? (green notes)
- What needs to be in place to achieve the objectives, like staff levels, physical environment, suitable indoor and outdoor facilities ..., i.e., *structure?* (yellow notes)

When the end of the listing seemed to be reached the facilitator asked “Have we really exhausted all problems and issues? At this point it is important that everything is on the table! Let´s take a short break and we will revisit the list when we are back.”

Intermediary stages

2 Qualitative causal loop diagram

At this point during the second meeting both the manager and participants expressed great satisfaction having come so far. They said that having worked through and discussed the priorities they had a shared sense of purpose that would guide them through their coming work.

When the notes had been rearranged and discussed, the facilitator asked which note was primary and should be the focus of a causal diagram that the group would be building together. They selected “satisfied residents and relatives”. Next, participants were asked which other notes had a direct influence on that objective. Those notes were moved closer to the focus note and the modeller gradually built a causal diagram by taking each note, discussing how it fitted into the causality, pasted it on the wall with an appropriate arrow showing the causality. The modeller felt slight frustration at this point as we had not uncovered any loop mechanisms, all was straightforward linear causality. He made a mental note that that

was my “problem”, it was not a problem for the participants as they were very happy about the causal diagram as it stood.

The modeller transformed the notes into a formal causal loop diagram using colour codes to distinguish between objectives, structure, and action. The diagram was introduced at the third meeting, discussed and minor amendments were made.

3 Qualitative causal loop diagram survey

At the third meeting participants were told that a questionnaire would be developed and sent the participants to investigate the causal diagram. The survey meant weighting all the inputs of the causal diagram and estimating the present rating of each variable in the diagram. The output was a revised causal diagram showing the relative weights using different line thicknesses and font sizes, which was presented and discussed at the fourth meeting to ensure that it was understood and shared by all (Figure 4). The group then reviewed the responses to the survey. The modeller then introduced a System Dynamics model to study the impact of changes on the main objective.

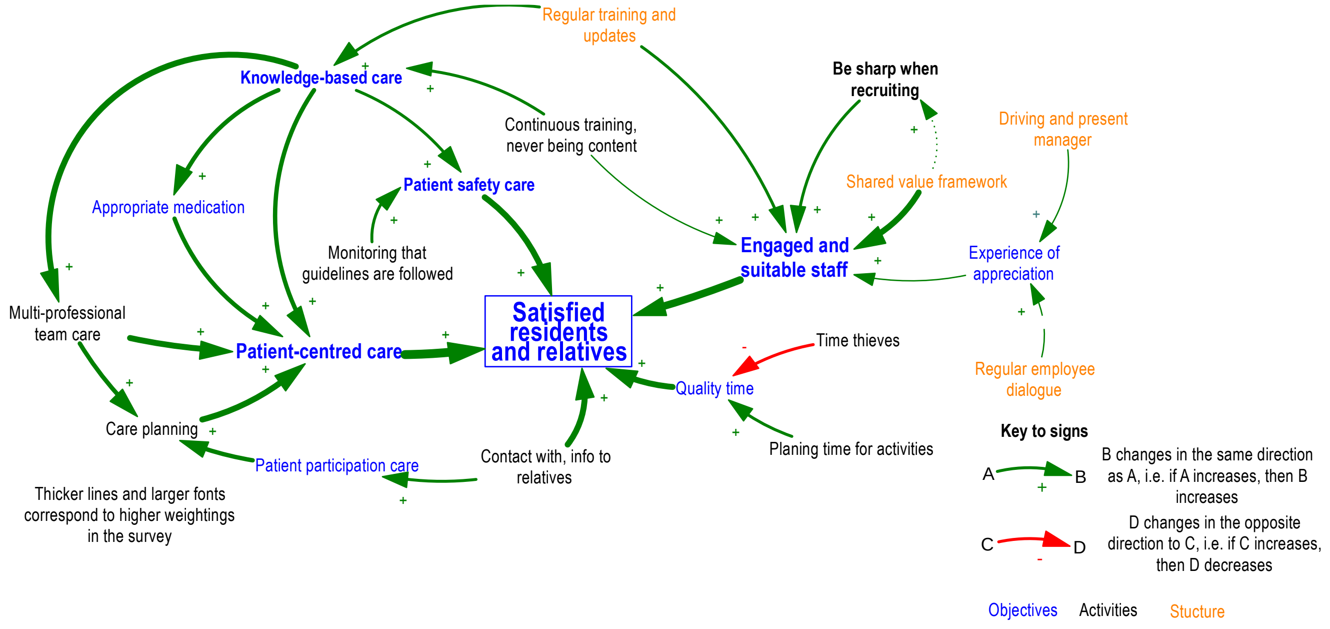

Figure 4 Case 3 - Weighted causal diagram exploring key factors in improving care for dementia patients

The purpose of the causal loop diagram was to form the basis of a stock and flow system dynamics model where the participants would be able to experiment with and understand the interaction between variables over time. The initial separation of factors during the problems and objectives inventory, into the three categories of objectives, activities and structure did not contribute at this stage. However, the weighting of factors indicated that clusters around engaged and suitable staff, patient centred care, knowledge-based care, and patient safety care were highly important in achieving the main objective of satisfied residents and relatives. Recruitment, training, shared values, and the role of the manager were found to be important factors for engaged and suitable staff. Knowledge-based care depended on training both as activity and structural factors. Patient-centred care was found to be a result of applied knowledge, medication, building on multi-professional teams both working and planning together as well as involving patients and relatives in the planning.

4 Action proposals

After the discussion of the causal loop diagram, Participants were asked, given this causality and the main objectives:

- what do we need to do more of?
- what do we need to do less of?
- what should we stop doing?
- what should we start doing?

The participants worked in smaller groups, wrote on large adhesive notes and presented their findings. The group found that they had uncovered a lot of relevant issues which were very meaningful for them.

They would continue the discussion at a staff meeting later in the year when they are to work with care philosophy, guidelines and work organisation. They saw clearly that they want to get better at individually adapting care through guidelines and individual care plans. The realised that the care plan is an important instrument in developing individual care. The group repeatedly returned to the necessity of good leadership and a manager who is present.

5 User interface

The model based on the qualitative causal loop diagram survey had a user interface so that participants could engage in discussions and testing which variables had the strongest effects and how long time it took to achieve change. One could then test the effects of individual or combined activities. An organisation cannot change all parameters at once, so the purpose of the model is to test and discuss where to start to achieve maximum initial effects.

Final stages

6 Scenarios, model simulation and experiments, workshop

Most of the fourth meeting was used experimenting with the model and “translating” suggested activities into variable settings and led to interesting discussions about priorities. Where should they begin when they reorganized?

7 Conclusions / action decisions

The purpose of the intervention was to move the group beyond traditional building programming and to engage them in an exploratory discussion of their work in the light of higher objectives. Although there was a rough plan at the outset, the detailed interventions were designed under way to facilitate a learning process.

The group was highly committed to the overall purposes and very engaged in their discussions. They found the process most useful and gained insights which they found very useful in their own process of redesigning their work. This allowed them to move from discussions of room functionality and square meters to an understanding of the work to be done in the rooms and thus having an entirely different discussion.

The prioritization process and the final model built on the survey results led to significant insights about everything not being equally important, but that some activities have more impact than others.

In particular the elderly care manager was highly satisfied over the contribution of the project in the undergoing change project.

Case 4 – Paediatric clinic

Background

The overall purpose of the project was to study the usability of System Dynamics to support pre-planning architectural processes. My brief was to use System Dynamics modelling to explore mainly qualitative, but also quantitative aspects of work processes to engage the group into considering development of work processes before specifying future needs of premises.

Here a paediatric clinic in a town with 25 000 inhabitants was studied. The clinic was housed at the local hospital, but organisationally was part of the major hospital in the district. The clinic in its turn had a small satellite unit in a small municipality. They considered their premises too small for their needs. A situation, which would be exacerbated as the physician at the satellite would be retiring and its patients be allocated to the main clinic.

The clinic had moved into its present premises in fifteen years earlier and it was not up to date with current requirements for hygiene, disease control and efficient patient processes. The number of patient visits to physicians and nurses respectively had increased over the years. In addition to this there were 1500 annual visits to the satellite clinic. Visits to welfare officers, dieticians and psychologists also had increased. More physicians in training also attended the clinic. All this had led to a shortage of workspace and examination rooms. Additionally, birth rates in the take-up area had increased, which would lead to even more visits in the future.

During the third meeting participants asked about the purpose and objectives of what the research group were doing. The participants described a history that led to a sense of resignation among staff. They had long stated that the facilities were unsatisfactorily, which had not been recognized neither by their main organisational unit at the regional hospital or the management of the local hospital where they were localized. They were concerned that the project would not be able help them with their dilemma. The leader of the research project described that the intent of the process was to surface their needs so that they could specify their needs of facilities. The participants accepted this and were willing to continue the work.

The issue resurfaced during the fourth meeting. It became clearer and clearer to the modeller that on one hand the group was very engaged in the work that was being done, but on the other hand their experience over the years of having their needs ignored meant that their motivation occasionally sagged.

Participants

Care unit manager, 2 paediatricians, 3 paediatric nurses, 1 medical secretary and 1 play therapist. In addition to this a research project leader, an architect, and a modeller.

Meetings were held in a tightly spaced room of the care unit. It was not easy to move about and put up adhesive notes on the wall. It was also difficult to physically emphasize who was leading or facilitating the meeting.

After the first meeting each meeting began with reflections since last. All meetings ended with reflections and summarizing who was supposed to do what before the next meeting

Start stage

1 Problems and objectives inventory

All participants briefly introduced themselves and the overall research project was presented at the first meeting. The group was divided into specialist sub-groups and were asked to write adhesive notes expressing their needs as objectives, e.g. “The clinic needs rooms to separate infected children in the waiting room and examination rooms”. Each group presented and discussions after each presentation.

The work was continued at the second meeting when all adhesive notes from the first meeting were put op on the wall again. Some initial notes had been slightly edited to conform to steering documents. The objectives were discussed at length and rephrased for clarity e.g., “Improve diagnostic precision” was changed to “High diagnostic precision” and “Reduce incorrect assessments” to “Correct assessments”. The group added an objective for a patient record system to be integrated into the care chain for higher patient safety.

When the end of the listing seemed to be reached the modeller asked “Have we really exhausted all problems and issues? At this point it is important that everything is on the table! Let´s take a short break and we will revisit the list when we are back.”

A subset of the group clustered the notes according to themes and the participants were allotted a small number of coloured adhesive dots to stick to the notes to indicate their personal priorities.

Intermediary stages

2 Qualitative causal loop diagram

After the action discussions at the second meeting the modeller began assembling a causal loop diagram, where the participants placed "infection control", "patient safety" and "person-cantered care" as the top priorities in the centre. The modeller explained the concept of direct causality and asked the group which of the other adhesive notes had a direct influence on the topic(s) in the centre. If the causality was not obvious to the modeller, he asked for an explanation and sometimes challenged it. In some cases, a note needed to be redefined by the participants. In this way a causal loop diagram was built interactively using many of the initial adhesive notes, sometimes adding new parameters to ensure clear causality. Causality was demonstrated by using arrow-shaped adhesive notes.

Prior to the third meeting the modeller converted the initial causal loop diagram from adhesive notes to a formal diagram to show such a diagram and explain the notation. At the third meeting the discussion of the objectives was revisited and then the meeting continued by building the causal loop diagram using the remaining adhesive notes.

Prior to the fourth meeting the modeller had prepared a set of PowerPoint slides that built up the causal loop diagram step-by-step. He worked through the diagram and kept asking if it was correct. The modeller made corrections as he worked through the presentation, adjusting names of variables as well as relations between variables.

3 Fact-finding

The modeller was relatively knowledgeable about how the regional hospital, to which the department belonged, worked with steering documents, planning and follow-up and requested relevant material. The research group got useful documents with population development, patient and staffing statistics etc. Given the concerns regarding the balance between patient volumes and capacity as well as the closure of the satellite it was clear that a simulation model combining quantitative data and qualitative aspects would be needed.

At the second meeting the modeller presented graphs of patient numbers and their future development. The numbers had been taken from a document regarding facility needs. Many participants felt that actual increases in patient visits were not accurately reflected in the data as visit registration was not fully reliable. Many visits to nurses had not been registered. The group discussed how to improve the data for a model.

The discussion continued during the third meeting. The questions from the modeller made it clear that the stated increase in patient volumes in relation to capacity was not clearly described in the steering documents etc. Visits were not recorded in a statistically useful way. When the clinic was built, the unit had 2000 annual visits to physicians and 900 visits to nurses, but when the project was carried totalled 7000 visits. Additional patient categories would be added in the future, such as rheumatic children, anesthetized patients waking up after simple surgeries etc.

4 Qualitative causal loop diagram survey

Causal loop diagrams can be very useful in understanding the interconnectedness of qualitative factors and which factors reinforce or balance outcomes. However, causal loop diagrams are "flat" in the sense that they do not allocate any weight to individual factors. At the fourth meeting participants were told that that a questionnaire to investigate the causal diagram was to be developed and sent to them. The survey meant weighting all the inputs of the causal diagram and estimating the present rating of each variable in the diagram.

The output was a revised causal diagram showing the relative weights using different line thicknesses and font sizes, which was presented and discussed at the fifth meeting to ensure that it was understood and shared by all (Figure 5). The responses to the survey were presented and discussed at the next meeting.

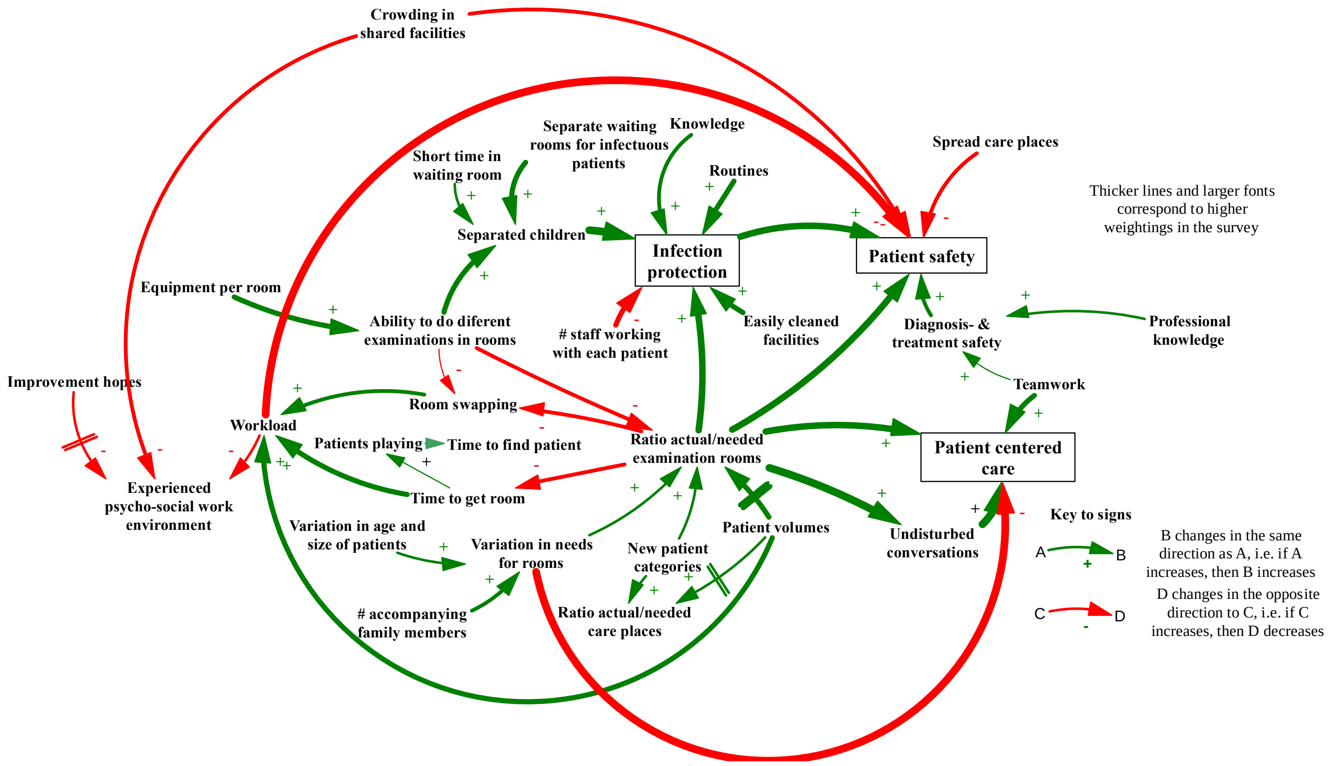

Figure 5 Case 4 - Weighted causal loop diagram exploring how to improve patient safety in a crowded paediatrics department

The purpose of the causal diagram was to form the basis of a stock and flow system dynamics model where the participants would be able to experiment with and understand the interaction between qualitative variables and their interactions with patient flows over time. Loops were briefly discussed, but not formally described. The causal loop diagram focused on clusters around the three major concerns for the participants (text in boxes in the diagram). Given the expected increase in patient volumes, crowding in waiting rooms and corridors could be expected as well as a shortage of examination and treatment rooms leading to waiting in corridors and room swapping. In turn, this could lead to infection risks as well as increased workloads. Paediatric patients usually have an accompanying parent, which can increase crowding given the already limited space. Patients' ages also span between 0-18 years, with highly different needs, presenting further challenges for the facilities.

5 Action proposals

At the second meeting the participants were divided into three groups to discuss activities in relation to the objectives:

- What do we need to do more of?
- What do we need to do less of?
- What should we start doing?
- What should we stop doing?

After putting up the adhesive notes each participant got a small number of coloured adhesive dots to put on the notes reflecting their personal priorities.

6 User interface

The model based on the qualitative causal loop diagram survey had a user interface so that participants could engage in discussions and testing which variables had the strongest effects and how long time it took to achieve change. One could then test the effects of individual or combined activities. An organisation cannot change all parameters at once, so the purpose of the model was to test and discuss where to start to achieve maximum initial effects.

The patient flow part of the model had variables for growth of patient volumes, closure of the satellite etc.

7 Problem visualization in model

The breakthrough understanding of the model and its explanatory value came when the patient inflow was doubled, patients were not separated and had to wait a long time – peak crowding. The group said that this was exactly how it had been the previous winter when retrovirus peaked. At that point, the group felt that the model was validated and spent quite some time experimenting with it.

Two questions around coping with crowding were discussed:

- How to equalize patient flows over a day
- The unit worked along two different patient physical paths. One corridor for out-patients and another for hospitalized patients, most of the latter were day-patients. Patient flows peaked at different times of the day for each corridor. Could patients be spread out over both corridors without losing overview?

Final stages

8 Scenarios, model simulation and experiments, workshop

The simulation model(s) were introduced in two steps. First a model of the qualitative aspects based on the causal loop diagram survey. The modeller introduced the interface of the stock and flow model, the initial state was based on the survey results and the concept of time to achieve change. He then ran some sample assumptions and changes after which he opened up for discussion and spent considerable time running the model according to the suggestions of the group.

9 Conclusions / action decisions

The participants were deeply engaged throughout the project despite the goal conflict mentioned above. Participants seemed to see that the results of the simulation could support their position as it clearly showed the effects of peak crowding and the satellite closure. In contrast to traditional planning which usually is based on perceptions of average patient flows the model showed criticalities. The group realized that this was an issue to be handled in the investment budget process.

Case 5 – accident and emergencies

Background

The overall purpose of the project was to study the usability of System Dynamics to support pre-planning processes. The brief of the modeller was to use System Dynamics modelling to explore mainly qualitative, but also quantitative aspects of work processes to engage the group into considering development of work processes before specifying future needs of premises.

Here an Accident and Emergency department was studied. They had about 50 000 patients per year and considered their premises too constrained to ensure proper patient safety. The premises were perceived as cramped and difficult to overview, risking patient safety. The hospital was embarking on a major review of the premises and the needs of the department.

Participants

Nine persons from the hospital took part: a facilities controller, the A&E manager, physicians, and nurses. In addition to this a research project leader, an architect, and a modeller.

After the first meeting each meeting began with reflections since last. All meetings ended with reflections and summarizing who was supposed to do what before the next meeting.

Start stage

1 Problems and objectives inventory

After general introductions and presentation of the overall research project at the first meeting. the participants were split into three sub-groups and used adhesive notes to describe problems, effects and what could be done. A person from each respective sub-group presented their conclusions were discussed in the full group. When the end of the listing seemed to be reached the modeller asked “Have we really exhausted all problems and issues? At this point it is important that everything is on the table! Let´s take a short break and we will revisit the list when we are back.”

A subset of the group clustered the notes according to themes and the participants were allotted a small number of coloured adhesive dots to stick to the notes to indicate their personal priorities.

There were major concerns about patient safety and throughput times. The premises were perceived as cramped and difficult to overview. Peak crowding and stasis could occur several times per week when staff felt out of control.

2 Qualitative causal loop diagram

After the action discussions at the second meeting the modeller began assembling a causal loop diagram, where “patient safety was placed as the top priorities in the centre. The modeller briefly explained the concept of direct causality and ask the group which of the other adhesive notes had a direct influence on the topic in the centre. The modeller elicited input from the participants connecting all other notes and made some adjustments on the way. Prior to the third meeting the modeller drew a formal causal loop diagram, which was presented at the meeting. The participants were divided into four groups to discuss the causalities and consider if anything was missing. The group reconvened and the modeller walked through the causal loop diagram in its entirety and some revisions were made.

3 Initial model

Prior to the second meeting the modeler had prepared a simple simulation model that explored the effects of variable patient inflows on throughput and waiting times. In reality this was handled by adding staff resource by bringing in physicians stationed at clinics and come to A&E in bursts. Increased variability was coped with by balancing the needs of patients in the clinic and in A&E. This led to a discussion about how in reality capacity was variable by bringing in additional specialist time from the hospital. It was also discussed how triage ensures that truly acute patients are handled as soon as possible.

4 Fact-finding

The initial model showed that a model combining both qualitative and quantitative aspects should be useful. However, data in budget and planning documents was not sufficiently fine-grained, so an additional fact-finding meeting where the modeller visited the department was decided on. Data was gathered on patient numbers by triage, department etc. as well as patient flows, staffing levels etc. As the premises were considered as cramped, the modeller also asked for an estimate of how many relatives etc. accom-

panied the patients. Estimates were about 1 person per 2 patients. This is an interesting factor to consider when planning space requirements. The number of patients in the system usually peaks at around 50, which means that there are another 25 in the rooms.

Given the opinion that too many patients come to A&E it is interesting to note that 40% of patients seen by a surgical specialist were admitted to the hospital and 55 % of those seen by a medical specialist.

5 Patient flow diagram

There was not sufficiently fine-grained data to draw a detailed Sankey-diagram of patient flows (Figure 6). The diagram served as a point of discussion and confirmation before moving into detailed modelling, which can be overly technical for participants. Generally, such a diagram is time efficient as any misunderstandings about flows are clarified before a complicated simulation model is built. The diagram created a sense among participants that the modeller “understood” their situation. The graph below was drawn to show rough flows.

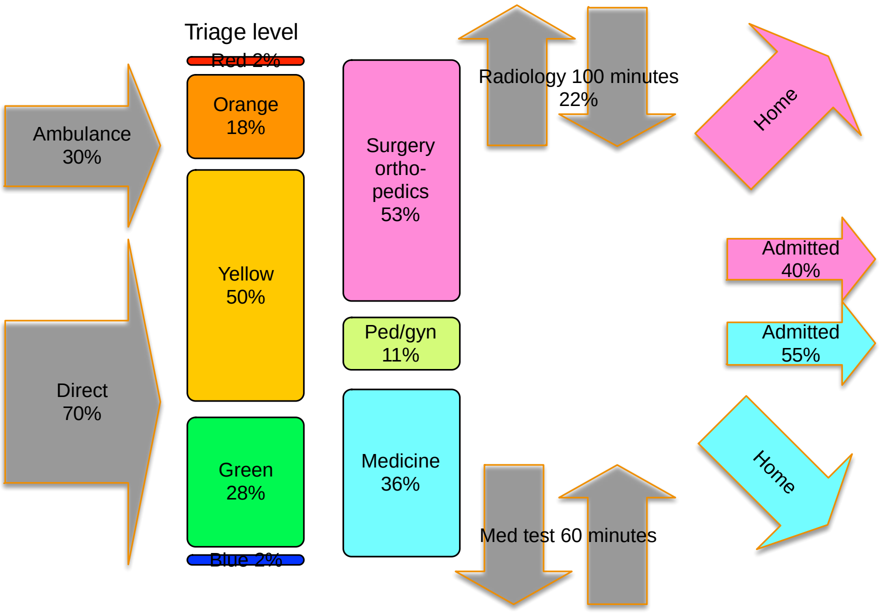

Figure 6 Case 5 – Patient flow diagram

6 Action proposals

At the second meeting the objectives stated during the first meeting were recapitulated, the participants were split into groups and they, given the objectives, were asked to list the following on adhesive notes:

- What they need to do more of
- What they need to do less of
- What they should stop doing
- What they should start doing.

7 User interface - Problem visualization in model

Based on the discussion of the introductory model presented at the first meeting and the fact-finding meeting the modeller extended the model to describe three patient groups.

- Red triage group, i.e., those who are taken care of immediately and given top priority over all other patients. This category was rapidly taken into surgery or admitted to the hospital for immediate treatment.
- Patients that needed lab testing or radiology, which meant that they were subjected to additional queues, first waiting for testing, then waiting for results and finally seeing a physician again.
- All other patients.

Studying the effects of this model highlighted that the additional queues for patients’ subject to testing/radiology can add substantial throughput time. As radiology closed between midnight and morning for all but critical patients this adds time as well.

A user interface was built, it can be pivotal for the participants in actively using the simulation model in their discovery process. It had a translative function between their reality and the actual simulation

model. The participants needed a basic understanding of the system dynamics model so that they could decide which variables they want to experiment with in the user interface and which outputs they want to see in graphs or tables to see the effects of their experimental decisions. Having a user interface directly involved the participants in asking "what-if" questions once the modellers have shown some experiments.

The simulation model and interface were introduced at the fourth meeting. The group raised a variety of questions:

- What if we can reduce waiting time for radiology and results to half?
- What if we can work with less than acute patients in different ways?
- If we can speed up throughput, the size of the waiting room could be halved.

The group worked through minor changes step by step and noted that each one did not have a very significant effect, but together improvement could be significant. Varying patient inflows had the greatest effects.

The major "aha" moment of the group came when they doubled the inflow, which led to long times in the waiting room and maximum crowding so that all areas were used for waiting. The reaction was "just like when everybody seemed to have retrovirus", "this is what it looks like when it is stasis". Several ideas were put forward for improving the model.

8 Qualitative causal loop diagram survey

Causal loop diagrams can be very useful in understanding the interconnectedness of qualitative factors and which factors reinforce or balance outcomes. However, causal loop diagrams are "flat" in the sense that they do not allocate any weight to individual factors. At the fourth meeting participants were told that that a questionnaire to investigate the causal diagram was to be developed and sent to them. The survey meant weighting all the inputs of the causal diagram and estimating the present rating of each variable in the diagram.

The output was a revised causal diagram showing the relative weights using different line thicknesses and font sizes, which was presented and discussed at the fifth meeting to ensure that it was understood and shared by all (Figure 7). The responses to the survey were presented and discussed at the next meeting.

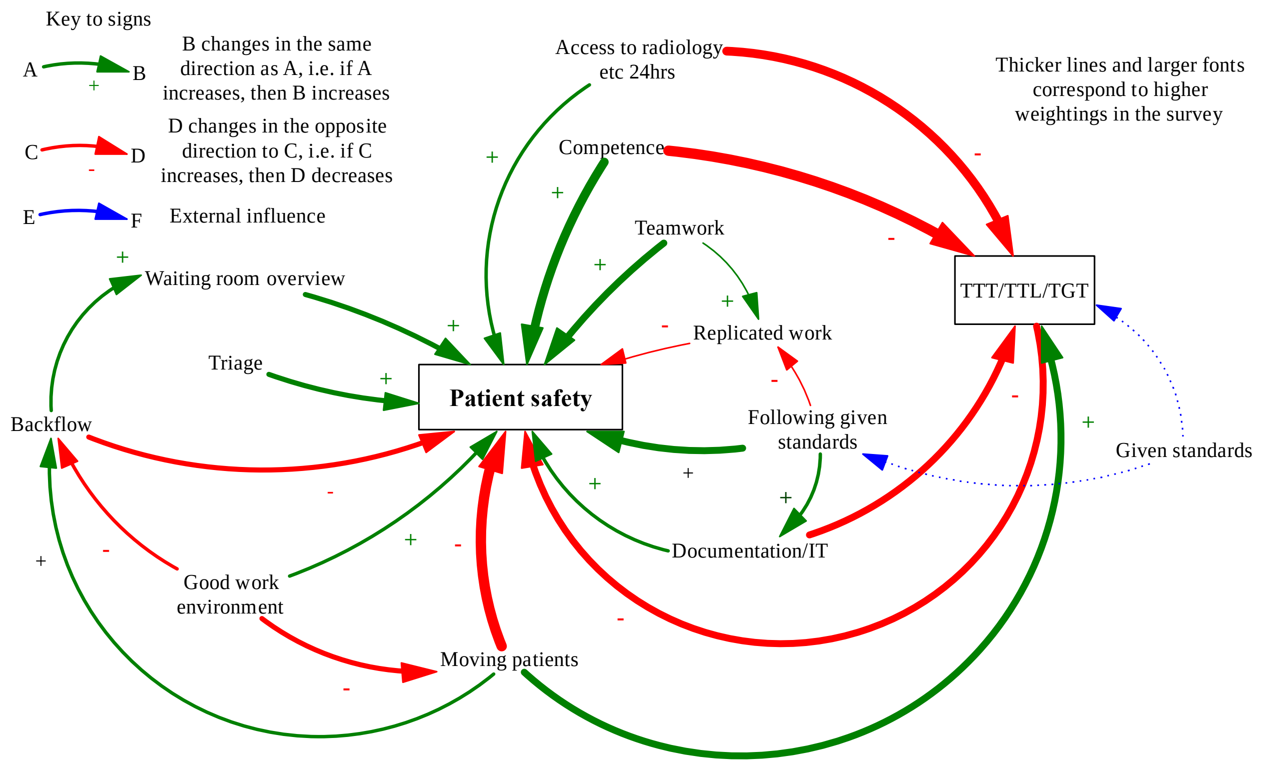

Figure 7 Case 5 - Weighted causal loop diagram concerning improving patient safety and length of stay in a crowded accident and emergencies department
(TTT = Time to triage, TTL=Time to see physician, TGT= Total length of stay)

Loops in the causal loop diagram were not explicitly identified or named as the intention was to include the key variables in a stock and flow diagram and to study the interaction with variations in patient flows. The causal loop diagram clustered around two factors, patient safety and time. Crowding in the acute wards was found to result in a backflow of patients to the waiting room and a loss of overview for the staff. High competence, standards, teamwork, and documentation were noted as important factors to avoid unnecessary rework, which negatively can affect patient safety and time in the system.

Final stages

9 Scenarios, model simulation and experiments, workshop

When the modeller walked through the weighted causal model, the response of the group was that it seemed realistic. The modeller demonstrated a few scenarios in the model then a series of runs based on suggestions by the group. Overall, the group considered that the model reflected reality.

- Increases in patient volumes led to increases in waiting and throughput times and a reduction in patient safety
- An increase in competence, with everything else constant, led to a slow increase in patient safety
- Allowing for medical testing and radiology around the clock led to a substantial increase in patient safety, maybe more than realistic
- Improving triage led to a small increase in patient safety
- A slight overcapacity allowed for coping with short peaks in patient inflow.

The group had a lengthy discussion about how they define triage, work environment and teamwork.

10 Conclusions / action decisions

The results confirmed the perceived issues around cramped facilities, but also illustrated patient flows peak every afternoon/evening while they have fairly constant staffing and that they needed to address how to handle the known parts of inflow variability. They also needed to consider how to faster move patients into regular wards when they are under pressure.

Some years later the department received entirely new and larger facilities after having done extended studies.
