# Supplementary material for: Insights Gained From a Re-analysis of Five Improvement Cases in Healthcare Integrating System Dynamics Into Action Research
Source: Int J Health Policy Manag. 2022 Feb 26;11(11):2707–18. doi: 10.34172/ijhpm.2022.5693 (PMC9818115; doi:10.34172/ijhpm.2022.5693)
Supplement: Supplementary file 2 — Analysis of Analytical Iterations. Descriptions of the major iterations of qualitative analysis. [file ijhpm-11-2707-s002.pdf]

Article title: Insights Gained From a Re-analysis of Five Improvement Cases in Healthcare Integrating System Dynamics into Action Research

Journal name: International Journal of Health Policy and Management (IJHPM)

Authors' information: Paul Holmström[1,2]*, Thomas Björk-Eriksson[2,3], Pål Davidsen[4], Fredrik Bååthe[5,6,7,8], Caroline Olsson[1,2]

[1]Department of Clinical Radiation Sciences, Institute of Clinical Sciences, Sahlgrenska Academy, Gothenburg University, Gothenburg, Sweden.

[2]Regional Cancer Centre West, Gothenburg, Sweden.

[3]Department of Oncology, Institute of Clinical Sciences, Sahlgrenska Academy, Gothenburg University, Gothenburg, Sweden.

[4]Department of Geography, University of Bergen, Bergen, Norway

[5]LEFO – Institute for Studies of the Medical Profession, Oslo, Norway.

[6]Institute of Stress Medicine, Gothenburg, Sweden.

[7]Sahlgrenska University Hospital, Gothenburg, Sweden.

[8]Institute of Health and Care Sciences, Sahlgrenska Academy, Gothenburg University, Gothenburg, Sweden.

(*Corresponding author: Email: paul@holmstrom.se)

Supplementary file 2. Analysis of Analytical Iterations

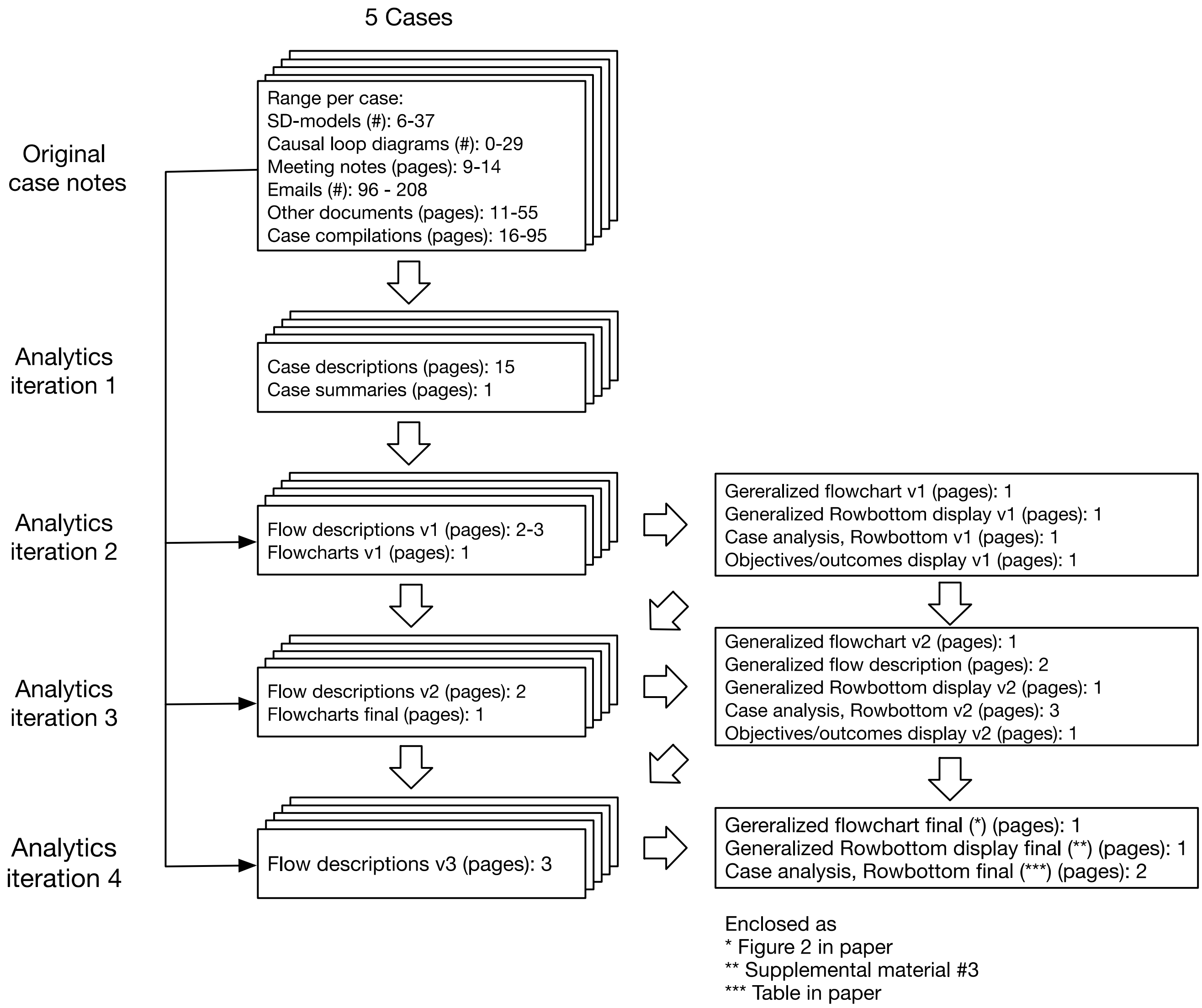

Figure 1 The four major iterations of analysis showing inputs and outputs by stage

An overview of the major iterations are shown in Figure 1. At each iteration, the analysis was first carried out individually by each co-author, then in group discussions, comparing and contrasting individual observations. Each stage led to insights and revised hypotheses that initiated a subsequent re-analysis of the original data and to revised displays. Throughout the iterations the original documentation was consulted to ascertain facts.

Documentary analysis

The primary documentary analysis was based on extensive material consisting of emails, meeting notes, causal loop diagrams, SD models etc. In two of the cases the background materials had been compiled into single documents, to test if the materials were easier to overview. This was still difficult to overview, and it was decided to instead aim for shorter case descriptions (final version as Supporting information 1).

Iteration 1

Input to the first iteration was longer case descriptions, from which short summaries were derived. Analysis showed that there were similar steps in all cases, but partially differently timed and executed. It was concluded that both similarities and differences needed to be demonstrated. It was decided to rework the descriptions so as to categorize work by meeting and to draw example workflow diagrams.

Iteration 2

The shorter case descriptions were further condensed into two-pagers, categorizing and describing work carried out during and between meetings. Example workflow diagrams were changed to show content blocks by meeting. The analytics team then deconstructed the flows by content and drew a generalized workflow diagram by content step, rather than by meeting. Interim hypothesises of work principles were discussed and it was concluded that Rowbottom's four questions could form the analytical structure to uncover actual work principles.The generalized description of Rowbottom's questions and their implications, from an earlier paper was revisited and formed the foundation for an initial analysis of each case by question. An interim summary table was drawn showing initial objectives and final outcomes by case. The team decided that both tables were promising and should be detailed further.

Iteration 3

Workflows by case were finalized and mapped onto the generalized flow, which was amended to be an "average" of the flows by case. The flow descriptions by case were revised according to the finalized naming of steps. The generalized Rowbottom description was amended to reflect learnings from the descriptions by case. The case-specific Rowbottom descriptions were amended for coherence between and within cases as well as with the objectives/outcomes tables. Key results and conclusions as described in the article began to emerge.

Iteration 4

The workflows by case were slightly corrected after the coherence checks in iteration 3. The table responding to Rowbottom's questions by stage was amended to include the information in the objectives/outcome table and again edited for coherence, after which minor associated adjustments were made to the generalized Rowbottom table. The framework for describing results and drawing final conclusions was in place. After the fourth iteration the authors had a sufficient and coherent structure to draw conclusions about work patterns and principles.
