# Supplementary material for: Insights Gained From a Re-analysis of Five Improvement Cases in Healthcare Integrating System Dynamics Into Action Research
Source: Int J Health Policy Manag. 2022 Feb 26;11(11):2707–18. doi: 10.34172/ijhpm.2022.5693 (PMC9818115; doi:10.34172/ijhpm.2022.5693)
Supplement: Supplementary file 3 — Rowbottom’s Four Socio-Analytical Questions. Rowbottom’s four socio-analytical questions and their implications for SD when working with groups to solve problems in work systems. [file ijhpm-11-2707-s003.pdf]

Article title: Insights Gained From a Re-analysis of Five Improvement Cases in Healthcare Integrating System Dynamics into Action Research

Journal name: International Journal of Health Policy and Management (IJHPM)

Authors' information: Paul Holmström[1,2]*, Thomas Björk-Eriksson[2,3], Pål Davidsen[4], Fredrik Bååthe[5,6,7,8], Caroline Olsson[1,2]

[1]Department of Clinical Radiation Sciences, Institute of Clinical Sciences, Sahlgrenska Academy, Gothenburg University, Gothenburg, Sweden.

[2]Regional Cancer Centre West, Gothenburg, Sweden.

[3]Department of Oncology, Institute of Clinical Sciences, Sahlgrenska Academy, Gothenburg University, Gothenburg, Sweden.

[4]Department of Geography, University of Bergen, Bergen, Norway

[5]LEFO – Institute for Studies of the Medical Profession, Oslo, Norway.

[6]Institute of Stress Medicine, Gothenburg, Sweden.

[7]Sahlgrenska University Hospital, Gothenburg, Sweden.

[8]Institute of Health and Care Sciences, Sahlgrenska Academy, Gothenburg University, Gothenburg, Sweden.

(*Corresponding author: Email: paul@holmstrom.se)

Supplementary file 3. Rowbottom's Four Socio-Analytical Questions

Table 1. Rowbottom's four socio-analytical questions and their implications for SD when working with groups to solve problems in work systems (direct quotes from Rowbottom in italics).

Rowbottom's stages of collaborative exploration in social analysis	Descriptions of stages from an AR perspective	Corresponding implications for SD
Starting point	The starting point is the problem that the group wants to study and address	Cases begin with an exhaustive group process inventory of problems and objectives
What is manifest?<br>How is it supposed to work?	*Discussion may start with a reference to any manifest statements (official reports, charts, memoranda, job descriptions and the like) which may exist.*<br>The group works through material that is relevant to the problem(s) to be addressed, exploring how the studied work system is intended to work.	A tentative SD model is built to reflect how the modeller perceives that the system is intended to work. The model will often fail, showing that what is manifest will not work as expected or that there are gaps in the knowledge underpinning the model.
Response	As the group explores manifest statements, the facilitator asks, "is this how it works in reality".	The modeller may say "I built a partial model on your data and my current understanding of your problem. The model does not seem to show reality as it is. What have I misunderstood or missed?"
What is assumed?<br>How do the participants believe it works?	*Discussion about participants' own assumptions about how things 'really' operate.*<br>Each participant shares their own perspectives on how things actually work. As they come from different professions or participate in different parts of a process, they are providing different perspectives of a whole.	Different participants will have different perspectives on what is missing in the model. This can be a highly collaborative phase where knowledge is shared, and the model iterated and revised rapidly. A user interface works as a "translator" between the group and the "language" of system dynamics,
Response	A unified view of how things actually work emerges through shared inquiry and striving to understand the perspective of all other participants.	Participants are satisfied of being heard and that their perspective, competence and knowledge is recognized. The user interface makes sense to

		them as it reflects the variables that they need to investigate.
What is extant?<br>How does it actually work?	*The situation as revealed by systematic exploration and analysis*<br>How things actually work have coalesced into a shared view of the actual problem or issue where the different views of participants complement each other rather than compete.	All perspectives come together in a final model that works in the sense that it behaves in ways that all participants expect. This can be a breakthrough point. Participants often say, "this is exactly how it is when …". In many cases, the initially stated problem has been redefined.
Response	When the group holds a shared view of how things actually work, then they are able to address the problems and issues at hand.	When the model has been validated to reflect history and the present as seen by the participants, it is used as an experimental tool in testing solutions to resolve problems and issues.
What is requisite?<br>How could it work?	*Discussion about how things could be clarified or improved; that is, to what is requisite.*<br>Based on the shared view of problems that need to be addressed the group can move on to suggesting actions, solutions, and shaping future policies.	Group discussions shape the use of the model in testing scenarios and selecting actions and policies that form a plan for the future.
