# Supplementary material for: Insights Gained From a Re-analysis of Five Improvement Cases in Healthcare Integrating System Dynamics Into Action Research
Source: Int J Health Policy Manag. 2022 Feb 26;11(11):2707–18. doi: 10.34172/ijhpm.2022.5693 (PMC9818115; doi:10.34172/ijhpm.2022.5693)
Supplement: Supplementary file 4 — Time Spent on Facilitation or Modelling. Estimation of share of time spent on facilitation or modelling by step in the generalized workflow where a combination of AR and SD was used to identify actionable solutions to problems in healthcare. [file ijhpm-11-2707-s004.pdf]

Article title: Insights Gained From a Re-analysis of Five Improvement Cases in Healthcare Integrating System Dynamics into Action Research

Journal name: International Journal of Health Policy and Management (IJHPM)

Authors' information: Paul Holmström[1,2]*, Thomas Björk-Eriksson[2,3], Pål Davidsen[4], Fredrik Bååthe[5,6,7,8], Caroline Olsson[1,2]

[1]Department of Clinical Radiation Sciences, Institute of Clinical Sciences, Sahlgrenska Academy, Gothenburg University, Gothenburg, Sweden.

[2]Regional Cancer Centre West, Gothenburg, Sweden.

[3]Department of Oncology, Institute of Clinical Sciences, Sahlgrenska Academy, Gothenburg University, Gothenburg, Sweden.

[4]Department of Geography, University of Bergen, Bergen, Norway

[5]LEFO – Institute for Studies of the Medical Profession, Oslo, Norway.

[6]Institute of Stress Medicine, Gothenburg, Sweden.

[7]Sahlgrenska University Hospital, Gothenburg, Sweden.

[8]Institute of Health and Care Sciences, Sahlgrenska Academy, Gothenburg University, Gothenburg, Sweden.

(*Corresponding author: Email: paul@holmstrom.se)

Supplementary file 4. Time Spent on Facilitation or Modelling

Almost all meeting time was spent on facilitated group discussions and most of the modelling work was conducted between meetings for all cases (Figure 1).

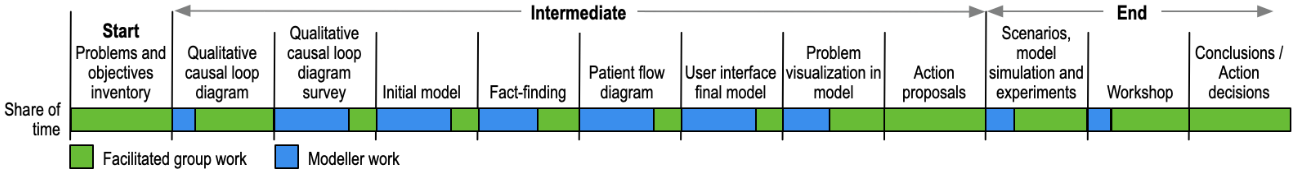

Figure 1 Estimation of share of time spent on facilitation or modelling by step in the generalized workflow where a combination of AR and SD was used to identify actionable solutions to problems in healthcare.
